# sgcocaller and comapr: personalised haplotype assembly and comparative crossover map analysis using single-gamete sequencing data

**DOI:** 10.1093/nar/gkac764

**Published:** 2022-09-15

**Authors:** Ruqian Lyu, Vanessa Tsui, Wayne Crismani, Ruijie Liu, Heejung Shim, Davis J McCarthy

**Affiliations:** Bioinformatics and Cellular Genomics, St Vincent’s Institute of Medical Research, 9 Princes Street, Fitzroy, Victoria 3065, Australia; Melbourne Integrative Genomics/School of Mathematics and Statistics, Faculty of Science, The University of Melbourne, Building 184, Royal Parade, Parkville, Victoria 3010, Australia; DNA Repair and Recombination Laboratory, St Vincent’s Institute of Medical Research, 9 Princes Street, Fitzroy, Victoria 3065, Australia; The Faculty of Medicine, Dentistry and Health Science, The University of Melbourne, Melbourne, Victoria 3010, Australia; DNA Repair and Recombination Laboratory, St Vincent’s Institute of Medical Research, 9 Princes Street, Fitzroy, Victoria 3065, Australia; The Faculty of Medicine, Dentistry and Health Science, The University of Melbourne, Melbourne, Victoria 3010, Australia; Bioinformatics and Cellular Genomics, St Vincent’s Institute of Medical Research, 9 Princes Street, Fitzroy, Victoria 3065, Australia; Melbourne Integrative Genomics/School of Mathematics and Statistics, Faculty of Science, The University of Melbourne, Building 184, Royal Parade, Parkville, Victoria 3010, Australia; Bioinformatics and Cellular Genomics, St Vincent’s Institute of Medical Research, 9 Princes Street, Fitzroy, Victoria 3065, Australia; Melbourne Integrative Genomics/School of Mathematics and Statistics, Faculty of Science, The University of Melbourne, Building 184, Royal Parade, Parkville, Victoria 3010, Australia

## Abstract

Profiling gametes of an individual enables the construction of personalised haplotypes and meiotic crossover landscapes, now achievable at larger scale than ever through the availability of high-throughput single-cell sequencing technologies. However, high-throughput single-gamete data commonly have low depth of coverage per gamete, which challenges existing gamete-based haplotype phasing methods. In addition, haplotyping a large number of single gametes from high-throughput single-cell DNA sequencing data and constructing meiotic crossover profiles using existing methods requires intensive processing. Here, we introduce efficient software tools for the essential tasks of generating personalised haplotypes and calling crossovers in gametes from single-gamete DNA sequencing data (sgcocaller), and constructing, visualising, and comparing individualised crossover landscapes from single gametes (comapr). With additional data pre-possessing, the tools can also be applied to bulk-sequenced samples. We demonstrate that sgcocaller is able to generate impeccable phasing results for high-coverage datasets, on which it is more accurate and stable than existing methods, and also performs well on low-coverage single-gamete sequencing datasets for which current methods fail. Our tools achieve highly accurate results with user-friendly installation, comprehensive documentation, efficient computation times and minimal memory usage.

## INTRODUCTION

Meiosis is a process required during sexual reproduction that generates gametes—egg or sperm cells—that contain half of the chromosomes of the parent cell (1). Chromosome segregation and meiotic crossovers create abundant genetic diversity in the offspring. Meiotic crossovers are also required for accurate chromosome segregation (1), and reduced crossover rates are linked to increased risk for trisomy 21 (2,3) and infertility (4,5). Past studies have shown that meiotic crossover rates and distributions vary greatly among species, sexes and even individuals (6–11). Variation in genetic factors, such as *PRDM9*, changes the distribution of crossover hotspots in human and mouse (6,8,12,13). Crossover-regulating genes also limit overall crossover rates (14–16). Populations of different demographic backgrounds show differences in recombination landscapes, for example hotspot locations differ in African-American genetic maps compared with those from Europeans and West Africans (17,18). It has also been shown that meiotic crossovers vary among sexes and crossover hotspots can be sex-specific (7,10,19).

Single-cell DNA sequencing of gametes collected from an individual can be used to construct personalised meiotic crossover distributions (9,11). The scalability of modern single-cell assays, especially droplet-based platforms, has made it possible to profile thousands of sperm cells per individual in one experiment (9). Assaying gametes from females is substantially more challenging at scale, but nevertheless technological developments will make large-scale single-gamete sequencing more common across sexes and organisms. The standard pre-processing pipeline of a single-cell DNA sequencing dataset generates a BAM (BinaryAlignment/Map) file that contains the mapped and cell-barcoded DNA reads from all single cells. Haplotyping these single-cell genomes with existing tools requires the steps of demultiplexing the single-cell reads according to their cell barcodes into thousands of intermediate files before applying haplotyping methods (20). While processing can be parallelised across cells, there are many opportunities to improve upon the use of methods developed for bulk DNA-seq data when analysing single-cell DNA-seq data by avoiding multiple reading and writing of millions of sequence reads. Even more importantly, there are substantial opportunities to improve on the accuracy of existing methods for haplotype reconstruction and genetic map construction from single-gamete data. Single-gamete DNA sequencing data usually have low depth of coverage across the genome per gamete, especially when generated using high-throughput droplet-based single-cell sequencing protocols. However with a group of gametes sequenced, the single nucleotide polymorphism (SNP) linkage in the gametes offers enough information for constructing chromosome-level phased haplotypes of the individual.

Therefore, we here introduce sgcocaller, an efficient command-line toolkit to directly process the large single-cell DNA-sequencing alignment files produced by the current standard pre-processing pipelines for personalised haplotype construction and single-gamete crossover identification. sgcocaller is also applicable to individual gametes sequenced by bulk DNA sequencing methods with minimal dataset pre-processing. We also introduce an associated Bioconductor/R package, comapr, for the construction, visualisation, and statistical analysis of crossover landscapes. Working in the Bioconductor ecosystem enables comapr to integrate seamlessly with other commonly used packages specialised in analysing biological datasets. Our new tools offer substantial improvements in accuracy of haplotype reconstruction from single-gamete sequencing data as well as greatly enhanced computational efficiency. With these tools, an easy-to-apply and end-to-end workflow for personalised haplotype assembly and comparative crossover map analysis has been made possible.

## MATERIALS AND METHODS

### Overview of sgcocaller and comapr

**Figure 1.**
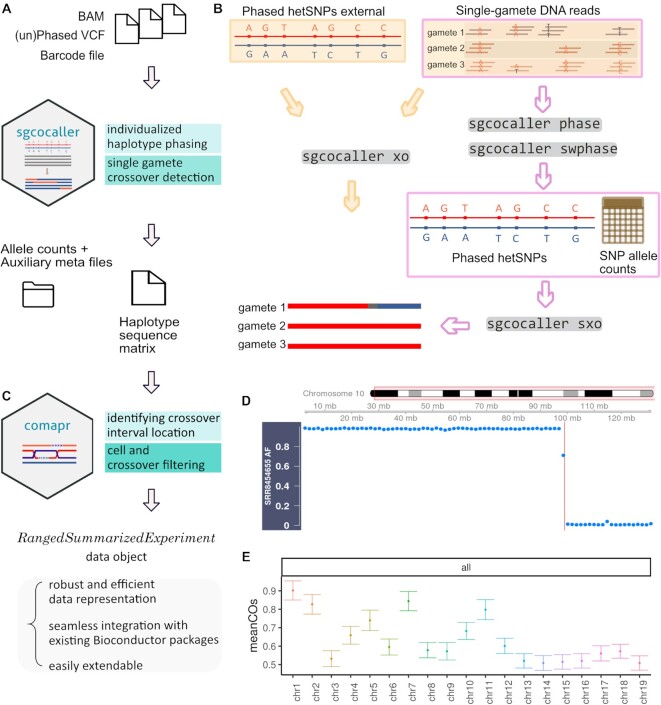
Overview of sgcocaller and comapr workflow including file and data flows of sgcocaller and comapr, as well as example plots generated by comapr. (**A**) sgcocaller takes the aligned DNA reads from all gametes, the list of hetSNPs (phased or unphased) and the cell barcode list and produces the single-gamete haplotype sequence in sparse matrix. (**B**) The diagram shows the main function of sgcocaller which is to resolve single gametes’ haplotypes from DNA reads using phased hetSNPs obtained from external sources (left) or from applying *sgcocaller phase* (right) by the two demonstrated workflows. (**C**) Output files from sgcocaller can be further processed and analysed by comapr, which identifies the crossover locations and filters false crossovers. It adopts the *RangedSummarizedExperiment* as the main data structure to enable seamless integration with existing Bioconductor packages. (**D**) Alternative allele frequencies plot for binned windows with crossover regions highlighted (vertical bar) from one selected gamete cell. (**E**) The mean crossovers per chromosome with error bars using crossovers called from the mouse sperm cells with the sgcocaller-comapr workflow. (both plots used the mouse sperm dataset (21)).

We have implemented a toolset for processing large-scale single-cell DNA sequence data from gametes with modules to construct the personalised haplotypes of the individual and to call crossovers for individual gametes. The toolset consists of two components, sgcocaller and comapr (see Data Availability), for complementary tasks. These packages have been implemented using appropriate programming languages (Nim and R) and released in multiple release formats to suit the expected use-case scenarios (Figure 1).

#### sgcocaller: single-gamete crossover caller

To suit the requirements of processing large alignment files containing DNA sequence read data for hundreds or thousands of individual gametes, sgcocaller has been designed as a command-line tool (CLT) implemented using the programming language Nim (*https://nim-lang.org/*). The software imports the *hts-nim* library (22) for fast processing of large DNA alignment files and variant calling files. It also imports the Rmath library (in programming language C from the R-project (R Core Team, 2021)) to use the well-defined distribution functions. To interface with the C-based library, a wrapper package (in Nim) was created and implemented as the *distributions* package that is openly accessible (see Data Availability). In crossover-calling scenarios with known phased haplotypes such as F1 hybrid samples generated by crossing known reference strains of species, and cases in which the haplotypes of donors are provided in an input VCF file, *sgcocaller xo*—the crossover calling module—can be applied directly. It requires three input files to call crossovers: the mapped BAM file with cell-barcoded DNA reads from all gametes, the VCF file of phased heterozygous SNP (hetSNP) markers and the list of cell barcodes (Figure 1A). When the phased haplotype information is not available from external sources, sgcocaller also offers a phasing module, *sgcocaller phase*, that is based on the SNP linkage data inherent in read data from the individual gametes to produce the personalised whole-chromosome haplotypes. To call crossovers using outputs of *sgcocaller phase*, *sgcocaller sxo* is recommended as it uses the intermediate files generated by *sgcocaller phase* including the phased haplotypes and the allele specific read count matrices as inputs, which avoids double handling of BAM/VCF files (Figure 1B). Our tool is engineered to operate directly on the output of common single-cell data processing pipelines such as CellRanger, STARSolo (23) and similar. With minimal pre-processing, sgcocaller can also work with bulk-sequencing samples as demonstrated in the application example with the mouse single-sperm dataset (see Application of sgcocaller and comapr to public datasets).

#### comapr: crossover analysis for construction of genetic distance maps in R

The second software component, comapr, serves as a post-processing tool that includes functions for finding crossover positions, quantifying crossover rates across groups, and conducting comparative analyses after the sequences of haplotypes are inferred by *sgcocaller xo* (or *sgcocaller sxo*) for each chromosome in gametes. It is implemented as an open-source Bioconductor/R package, which offers easy integration with other packages in Bioconductor for analysis of biological data and statistical testing. comapr includes functions to directly parse output files generated by *sgcocaller xo* or *sgcocaller sxo* with tunable parameters to systematically filter out potential false positive crossover calls and create structured data objects to represent the crossover information across all cells (Figure 1C). comapr provides quality checking functions and visualisations to understand the features of the underlying dataset and for choosing sensible filtering thresholds. It enables convenient plotting of summary plots such as number of crossovers per sample group, converting crossover rates to genetic distances in units of centiMorgans, and plotting genetic distances over chromosomes or the whole genome (see Application of sgcocaller and comapr to public datasets). comapr integrates with the genomic visualisation package Gviz (24) and easily generates alternative allele frequency plots with crossover intervals highlighted or with genomic feature tracks overlaid on top of identified crossover tracks (Figure 1D, E). To facilitate easy statistical significance testing for comparisons of crossover rates in gamete groups, such as gametes collected from individuals with different genetic backgrounds or from different experimental groups, comapr implements two re-sampling based methods, bootstrapping and permutation testing.

### *sgcocaller phase* and *sgcocaller swphase*

**Figure 2.**
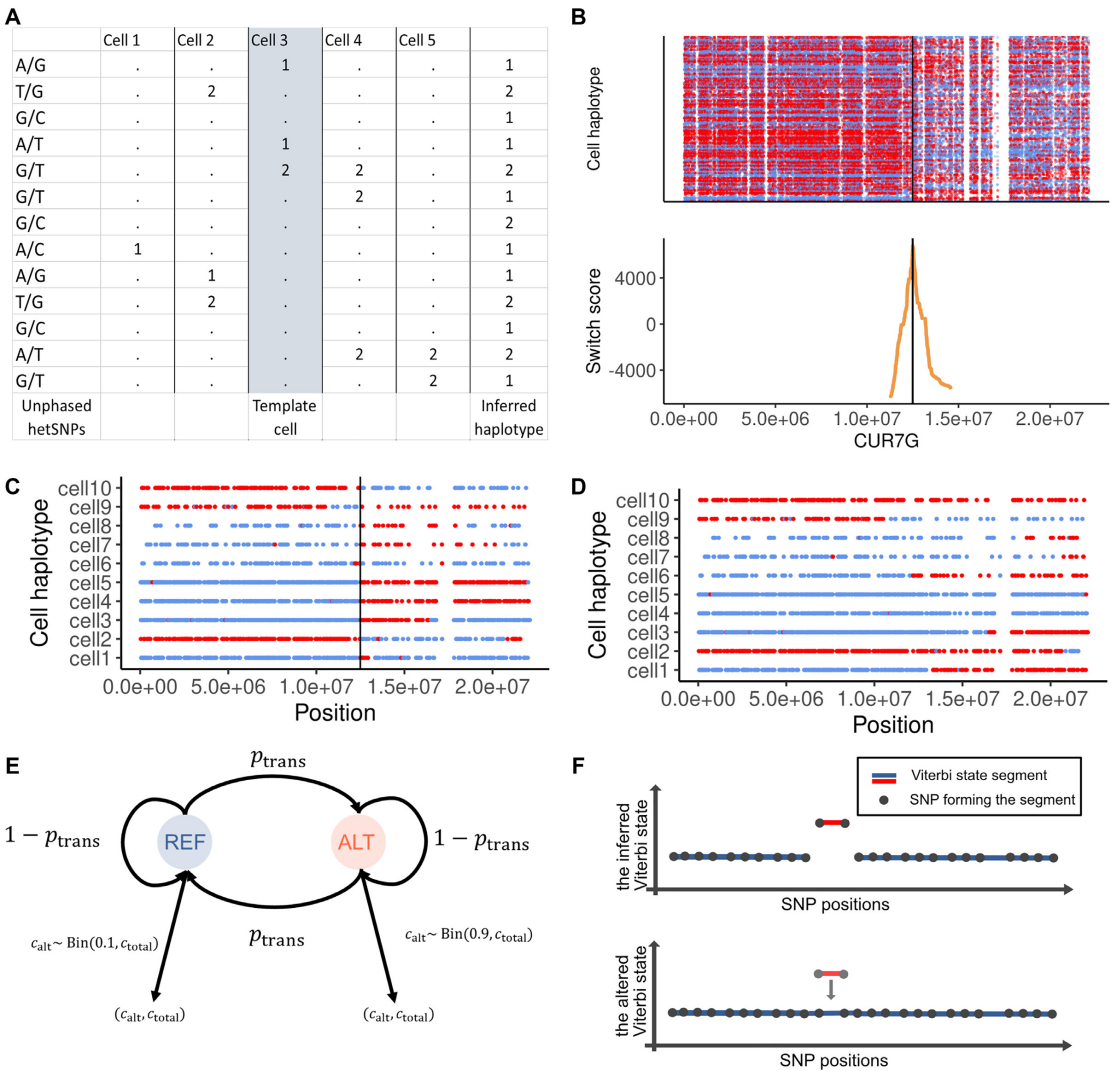
*sgcocaller phase* and *sgcocaller swphase* generate personalised haplotypes from single-gamete data. (**A**) Genotype matrix of an example of five gametes regarding the list of the hetSNPs with the chosen template cell highlighted. Template cell’s genotype sequence is used as the backbone for generating the inferred personal haplotype. (**B**) (Top) Cell genotypes along the list of SNPs were plotted and colored by which haplotypes they were matched with (the inferred template haplotype or its complimentary haplotype). (Bottom) Switch scores were calculated and plotted for SNPs in the inferred haplotype sequence, which were based on the genotypes of the flanking SNPs across all cells. The switch error position was found as the identified peak in switch scores (the black line). (**C**, **D**) Genotype sequences of ten cells were plotted and colored by whether the SNP’s genotype matching the inferred template haplotype or its complimentary haplotype for the case when there was a switch error in the inferred haplotype with the error position indicated by the black vertical line (C), and when there were no switch errors (D). Plots in b,c,d were generated using the apricot gametes dataset (20). (**E**) Diagram of the two-state (REF and ALT) hidden Markov model implemented in *sgcocaller xo*. The two possible alleles at each hetSNP site can be referred as REF or ALT arbitrarily. Binomial distributions have been adopted for modelling the relationship of observed allele counts and the underlying haplotype states of hetSNPs. The transition probability between two states (*p*_trans_, the distribution parameters (e.g., 0.1, 0.9) are configurable options when running *sgcocaller xo* (see Sgcocaller xo and sgcocaller sxo). (**F**) Diagram of the Viterbi state segment with the inferred hidden states (output from *sgcocaller xo*) and the altered hidden states (artificially generated by reversing the inferred states of the SNPs in the underlying segment). Segments are colored by their states. Dots represent the SNPs forming the segments.

Detecting crossovers in the gametes’ genomes requires the haplotypes of the individual’s genome. When the individual’s haplotypes are not available from other sources, sgcocaller offers a module *sgcocaller phase* that is able to produce the phased hetSNPs from unphased hetSNPs using the available single-gamete data (Figure 1A). *sgcocaller phase* uses SNP markers’ co-appearance information across all gametes to generate the chromosome-scale haplotype of the individual from single-gamete data, an idea that has also been applied in a previous study (25) (Figure 2A). Cells in the context of this manuscript are haploid gametes unless otherwise specified.

To increase the algorithm’s efficiency and fully utilise the known biological mechanisms of meiosis and meiotic crossovers, when phasing each chromosome the phasing algorithm in *sgcocaller phase* first finds a template cell (a cell putatively has not inherited crossovers with respect to the chromosome) and uses the cell’s genotype sequence along the chromosome as the template haplotype for the chromosome (Figure 2A; see Supplementary Methods). With respect to each chromosome, genotype sequences of paired cells are compared to find a pair of cells with the same genotype sequences. Such cells are candidates for template cells (see Supplementary Methods). Our approach next fills in the missing SNPs in the template haplotype using SNP linkage information from other gametes to increase the completeness of the phased haplotypes. The inference of missing SNPs is based on the fact that meiotic crossovers are low frequency events across chromosomes and crossover positions are sparse. The SNP linkages in small chromosome regions across all haploid gametes are therefore reliable for reconstructing the donor’s haplotypes (see Supplementary Methods).

To avoid relying on finding ideal template cells, which may not exist for certain gamete populations, for generating corrected phased haplotypes, we have included a switch error correction module *sgcocaller swphase*. When an ideal template cell is not used for phasing in the previous step, the chosen template cell may have crossovers leading to switching errors in the inferred haplotype. *swphase* produces switch scores (inspired by the block splitting function in HapCUT2 (26)) for a selected list of SNPs with a higher risk of having switch errors and identifies the switch positions to generate the corrected haplotype (Figure 2B; see Supplementary Methods).

The two modules (*phase* and *swphase*) can be applied in one step by calling *autophase* which runs *phase* and *swphase* subsequently. In application scenarios where ideal template cells are expected, users can choose to run *phase* only and save computational time. *sgcocaller phase* also generates auxiliary files for conveniently plotting diagnostic plots that help to inspect the quality of the inferred haplotypes and check for switch errors (Figure 2C). Since crossovers are low frequency events at each site across the gametes, switch errors in the inferred haplotypes can be identified by inspecting the diagnostic plot. When the plotted genotype sequences of all cells show existence of crossovers at the same position, it indicates a ‘crossover’ or switch error has occurred in the inferred haplotype (Figure 2C, D).

### *sgcocaller xo* and *sgcocaller sxo*

The crossover calling module *sgcocaller xo* infers the haplotype states in the haploid genomes of gametes. Two haplotypes (represented by the list of red or blue alleles on each chromosome in Figure 1B) can be found in the diploid genomes of individuals and the gametes from the individual inherit a combination of the two haplotypes (Figure 1B). Gametes have haploid genomes, therefore theoretically there is only one type of allele that can be observed at each SNP position in each gamete. However, due to technical noise such as mapping artefacts, there can be a substantial number of SNP sites with two types of alleles found but with biased ratio towards the true allele type in the genome. To suppress the potential noise in the dataset, *sgcocaller xo* implements a two-state hidden Markov model (HMM) and adopts binomial distributions for modelling the emission probabilities of the observed allele read counts (Figure 2E; see Supplementary Methods).

With this HMM, the commonly used dynamic programming method, the Viterbi algorithm (27), is implemented in *sgcocaller xo* to solve for the most probable hidden state sequence for each chromosome in each cell. Since the two hidden states in the HMM represent the haplotype origins of DNA segments (represented by allele types of a list of SNPs) in the gametes’ genomes, the transition from one state to another in the hidden state sequence between two SNP markers corresponds to a crossover detected. As currently designed, sgcocaller is intended to work on data of gametes collected from diploid individuals. The *sgcocaller sxo* module supplements the crossover calling module *sgcocaller xo*. It runs the same core function as *sgcocaller xo* but uses the generated outputs (i.e., allele count matrices and the phased haplotypes) from *sgcocaller phase* instead of the original BAM and VCF files, thus eliminating unnecessary double handling of large DNA sequencing data.

In addition to applying the HMM to the allele counts and using the Viterbi algorithm for inferring the hidden state sequences, we also included calculation of a crossover confidence score, which is the log-likelihood ratio of the Viterbi state segment. The log-likelihood ratio of a segment is derived by finding the difference between the log-likelihood of the segment given the inferred state and the log-likelihood of the segment given the altered state (log-likelihood of the first path given the data minus the log-likelihood of the second path; Figure 2F; see Supplementary Methods).

### Scope of sgcocaller

Although sgcocaller has been optimised to work on barcoded large-scale single-cell DNA sequencing data with outputs in formats of sparse matrices, it can also—with simple preparation—be applied to sequencing reads from one cell or bulk DNA sequencing samples for which the cell barcodes are not available. We released open-access tools for preparing the single-gamete DNA sequencing datasets generated using bulk-like protocols (individual sequencing libraries that generated separate sets of reads for gametes) and provided examples of applying sgcocaller on such datasets (21,28) (see Application of sgcocaller and comapr to public datasets).

### comapr: implementation

#### Data structure

The ‘RangedSummarizedExperiment’ class defined in the Bioconductor R package ‘SummarizedExperiment’ (Morgan *et al.* 2020) is used as the main data structure in comapr for storing the SNP interval regions and the number of called crossovers per cell (Figure 1C). The data slot ‘rowRanges’ is used for storing the SNP intervals, whereas the cell-level information is stored in the ‘colData’ slot. Using ‘RangedSummarizedExperiment’ also enables comapr to conveniently integrate with the various genomic coordinate plotting functions in the Gviz package (29).

#### Resampling-based functions for testing differences in crossover profiles

To test for differences in the number of crossovers between any two groups of cells, we have implemented re-sampling methods in comapr, specifically permutation and bootstrapping tests (30–32). The two resampling-based functions in comapr are able to either calculate empirical *P*-values (permutation testing) or bootstrap confidence intervals for the estimate of the group differences (see Supplementary Methods).

### Pre-processing public datasets

#### Mouse sperm dataset

Raw fastq files of 217 mouse sperm cells were available from accession GSE125326 in the Gene Expression Omnibus (21), and fastq files of 194 mouse sperm cells were downloaded. The downloading process of the rest 23 cells failed and they were not analysed. We applied fastp-v0.20.1 (33) on the raw reads for filtering out low quality reads and adapter trimming, before applying minimap2-2.7_x64-linux (34) for aligning the reads to the mouse reference genome mm10. The mapped reads were further processed by GATK MarkDuplicates and GATK AddOrReplaceReadGroup from the GATK-v4.2 pipeline (35). Read sorting and indexing were performed using samtools-v1.10 (36,37). A customised open-access tool appendCB (see AVAILABILITY OF DATA AND MATERIALS) was applied to add barcode sequences to each sperm’s DNA reads with tag CB using its SRR sequence. The DNA reads in each sperm sample were sub-sampled to retain half of the reads and merged into one barcode-tagged large BAM file that was analysed in this study as the mouse sperm dataset. The merging and indexing of BAM files were achieved using samtools-v1.10 (36).

We followed the main steps described in the original study for finding heterozygous SNPs in the mouse donor’s genome (21). We first called variants *de novo* on the bulk sperm sample ‘SRR8454653’ (sequenced DNA reads of pooled multiple sperm) using GATK-HaplotypeCaller. Only the hetSNPs with mapping quality score larger than 50, and depth of coverage within the range of 10–80 were kept (MQ > 50and DP > 10 and DP < 80). Since the mouse donor was an F1 hybrid (C57BL/6J X CAST/EiJ), the list of reference hetSNPs was downloaded (CAST_EiJ.mgp.v5.snps.dbSNP142.vcf.gz and C57BL_6NJ.mgp.v5.snps.dbSNP142.vcf.gz) from the Mouse Genome Project (38). The called variants in sample SRR8454653 were further filtered to only keep the positions which were called as homozygous alternative (GT==1/1) in CAST_EiJ.mgp.v5.snps.dbSNP142.vcf.gz and not overlapping with variants in C57BL_6NJ.mgp.v5.snps.dbSNP142.vcf.gz. Scripts are publicly available (see Data Availability).

#### Mouse sperm low coverage dataset

To generate a mouse sperm dataset that mimics the coverage level of a typical single-gamete DNA sequencing dataset (e.g., apricot gamete dataset), the DNA reads from the merged mouse sperm BAM file (as described before in section Mouse sperm dataset) were sub-sampled using samtools-v1.10 (36) to a fraction of 0.15 to yield the further low coverage mouse sperm dataset (msperm_low-coverage).

#### 10X scCNV apricot gametes

Two experiments were conducted to sequence the apricot gametes in the original published study (20). The pre-aligned BAM files (of two experiments) were downloaded from European Nucleotide Archive (ENA) under accession number ‘PRJEB37669’. The downloaded pre-aligned BAM files were converted to fastq reads with samtools-v1.10 (36). To keep the cell barcode information for each DNA read in the converted fastq files, the cell barcode sequence was appended to each fastq read’s sequence name. Reads in fastq files were then mapped to the published haploid genome ‘Currot’ using minimap2-2.7_x64-linux (34). The identification of hetSNP markers was performed by running bcftools-v1.10 on the pooled DNA reads from the two experiments. The identified hetSNPs were filtered using the same command as from the original study(20) (QUAL > 200 & FORMAT/DP < 280 & FORMAT/DP > 120 & FORMAT/GT==’0/1’ & (FORMAT/AD[0:1])/(FORMAT/DP) > 0.38 & (FORMAT/AD[0:1])/(FORMAT/DP) < 0.62). These filtering settings mean we only kept the SNPs with quality score larger than 200, total depth of allele coverage smaller than 180, genotypes as heterozygous, and the alternative allele frequency is in the range of 0.38-0.62. Gametes from the two experiments were merged in the analysis and gametes with barcode collisions were removed. Barcodes from 367 gametes were kept. Scripts are publicly available (see Data Availability).

#### Human sperm dataset

Raw fastq files of 11 sperm cells from a human individual were downloaded from the NCBI Sequence Read Archive (SRA) with SRR accessions provided in the original study (28). Like the the processing steps for mouse sperm dataset, we used fastp-v0.20.1 (33) on the raw reads for quality controlling fastq reads. minimap2-2.7_x64-linux (34) was run for aligning the reads to the human reference genome hg19. MarkDuplicates and AddOrReplaceReadGroup from the GATK-v4.2 pipeline (35) were applied. Finally, the BAM files of individual sperm cells were merged after adding CB tag by appendCB (see Data Availability).

### Phasing 10X scCNV apricot gametes

To test the phasing performance of sgcocaller, the 367 gametes from the apricot dataset (20) were used for generating the phased haplotype for the apricot sample. The haplotypes generated by sgcocaller were compared with the published assembly ‘Currot’ (see Supplementary Methods).

### Calling crossovers

We applied sgcocaller and comapr on public datasets to demonstrate the application of the software tools (see Application of sgcocaller and comapr to public datasets). The application examples cover different use-case scenarios: (i) a mouse sperm dataset (21) for which the haplotypes of the donors were known and (ii) the apricot gamete dataset with haplotypes of the donor inferred by *sgcocaller phase* before calling crossovers using *sgcocaller xo*. For the apricot dataset, crossover results when using the phased hetSNPs obtained from the published study (20) were also generated using *sgcocaller xo* to compare the differences in crossover calling results between using the haplotypes inferred by *sgcocaller phase* and the known haplotypes from the original study (see Supplementary Methods).

Crossover results were obtained by further processing the output files from *sgcocaller xo* and *sgcocaller sxo* (see Supplementary Methods) on different datasets using functions from comapr including cell filtering, false positive crossover filtering and genetic distances calculation and visualisation (see Supplementary Methods and public code repositories Data Availability).

### Phasing performance comparison

Performance of *sgcocaller phase|swphase* was compared to Hapi (39) on the human sperm dataset (28), the apricot gametes generated by the 10X scCNV protocol (20), the mouse sperm dataset (21) and the mouse sperm dataset with low read coverage (see Results: sgcocaller advances performance and efficiency). Exact options and parameters for running the two methods on the testing datasets are included in the Supplementary Methods. For each set of gametes from each dataset, 10 dataset repeats were constructed by leaving out 10% of the gametes except for the 11 human sperm cells where 11 dataset repeats were constructed. For the human sperm dataset, the list of unphased hetSNPs and phased results (used as ground truth) were downloaded from the supplementary dataset shared in the published study (28). For the apricot dataset, the haploid genome assembly ‘Currot’ published previously was used as the haplotype ground truth. The known haplotypes of the known mouse founder strains were used as the ground truth for the mouse sperm (and mouse sperm low coverage) datasets.

## RESULTS

### sgcocaller generates highly accurate phasing results

**Figure 3.**
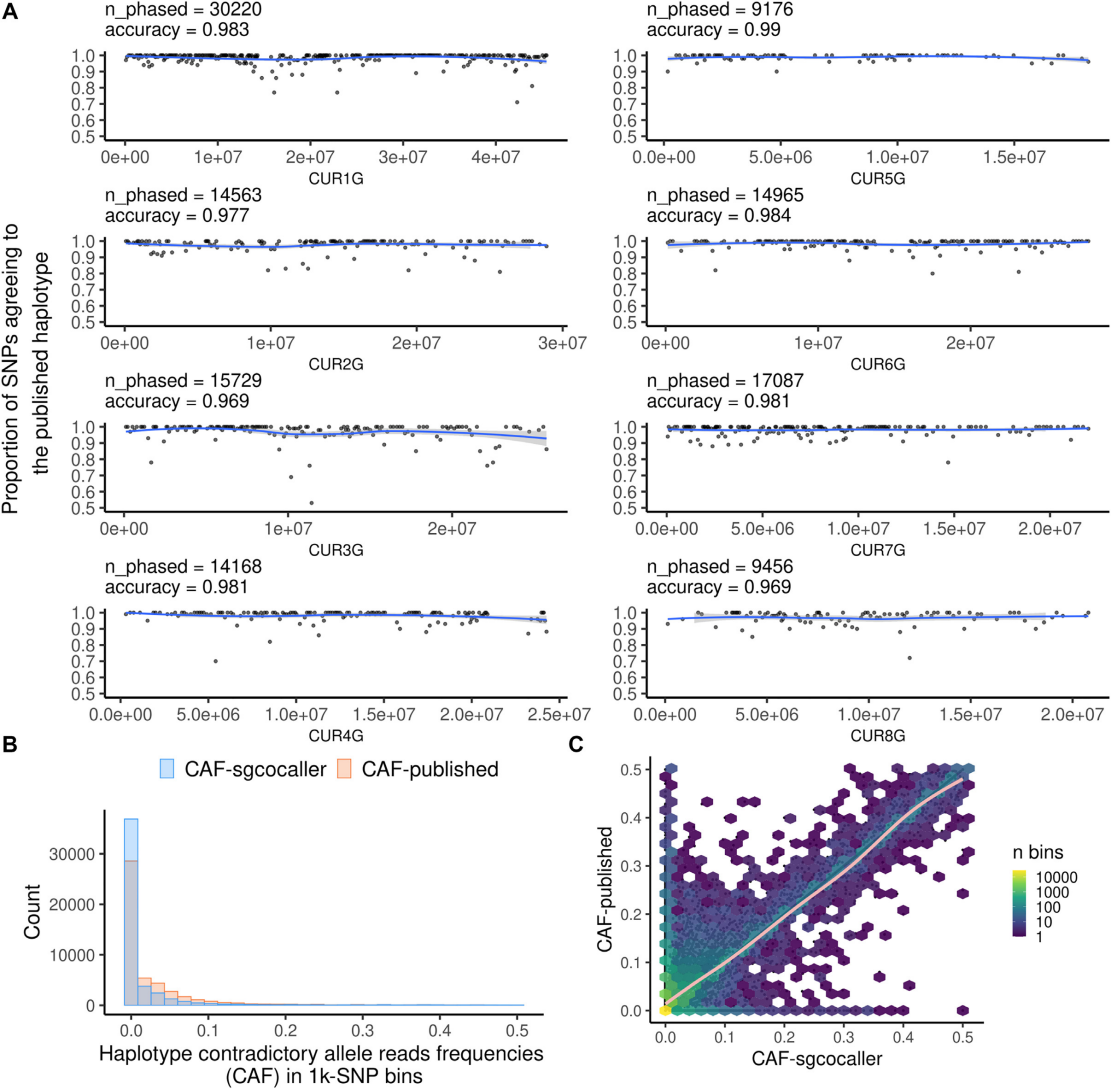
Comparisons between haplotype inferred by sgcocaller phase versus the published haplotype using the apricot dataset (20). (**A**) Proportion of SNPs agreeing with the published haplotype in 100-SNP bins across eight chromosomes from the using the apricot dataset (20). The number of phased SNPs (*n_phased*) by *sgcocaller phase* and phasing accuracies (*accuracy*) were printed on top of each panel. Smoothed curves were fitted by using ‘loess’ method (40) from R (R Core Team, 2021). (**B**) The histogram of haplotype contradictory allele read frequencies (CAFs) for bins of 1000 SNPs were plotted from all chromosomes colored by results using the haplotype generated by *sgcocaller phase* (CAF-sgcocaller) or the published haplotype (CAF-published). More bins with CAF valued at zero when using the haplotype by *sgcocaller phase* suggested that the haplotype from *sgcocaller phase* was more concordant with the single-gamete data. (**C**) The two alternative read frequencies (CAF-sgcocaller and CAF-published) for each bin were plotted in scatter plot with CAF-sgcocaller on the x axis and CAF-published on the y axis. Hexagons were plotted overlaying the scatter plot with color scale indicating counts of bins covered by the hexagon area. Fitted curve that aligned with the diagonal line was plotted using the method ‘gam’ (41) from ggplot2 ((40);R Core Team, 2021). Alternative allele in these plots are always the alleles with fewer read counts in each bin.

We applied *sgcocaller phase* (for generating haplotypes) and *sgcocaller swphase* (for identifying and correcting switch errors in the inferred haplotypes) on an apricot pollen dataset generated (see Materials and Methods) with the 10X scCNV protocol (a droplet-based single-cell DNA sequencing protocol) (20). Although *sgcocaller swphase* can be run selectively and only for cases when switch errors are identified in the inferred haplotype by *sgcocaller phase*, we applied both modules on all chromosomes. We refer to the final phasing results as generated by *sgcocaller phase* although *sgcocaller swphase* has been applied (see Supplementary Methods). The phasing results of *sgcocaller phase* demonstrate very high concordance with the haplotypes from the published assembly from the same study (Figure 3A, B). To evaluate performance of the phasing module in sgcocaller, the phasing accuracy was calculated using the fraction of concordant hetSNPs between the haplotype inferred from *sgcocaller phase* and the published haplotype. The hetSNPs were grouped into bins with 100 consecutive SNPs in each bin. The proportion of SNPs matching the published haplotype in 100-SNP bins concentrated at 1.0 across eight chromosomes indicating high phasing accuracy across the genome (Figure 3A and Supplementary Figure S1). We defined the haplotype contradictory alleles as the alleles in the haplotype sequence (e.g., columns in i, Supplementary Figure S2) supported by fewer DNA reads in each 1000-SNP bin in each gamete, and summarised the haplotype contradictory allele read frequencies (CAF) in 1,000-SNP bins across gametes. With this approach, we found that using the haplotypes generated by *sgcocaller phase|sgcocaller swphase* resulted in more bins with CAF valued at zero than using the published haplotype (Figure 3B, C). These results suggest *sgcocaller phase|sgcocaller swphase* generated haplotypes that are more concordant with the single-gamete read data than the published assembly. We note that these results do not directly imply the haplotypes by sgcocaller are more accurate than the published assembly but rather simply that the haplotypes inferred by sgcocaller match the observed single-gamete data better. However, there might be errors in SNP calling in the single gametes due to technical issues (e.g. base calling errors, mapping artefacts) and the low depth of read coverage at each SNP.

### Application of sgcocaller and comapr to public datasets

#### Bulk DNA sequencing dataset of single mouse sperm cells

**Figure 4.**
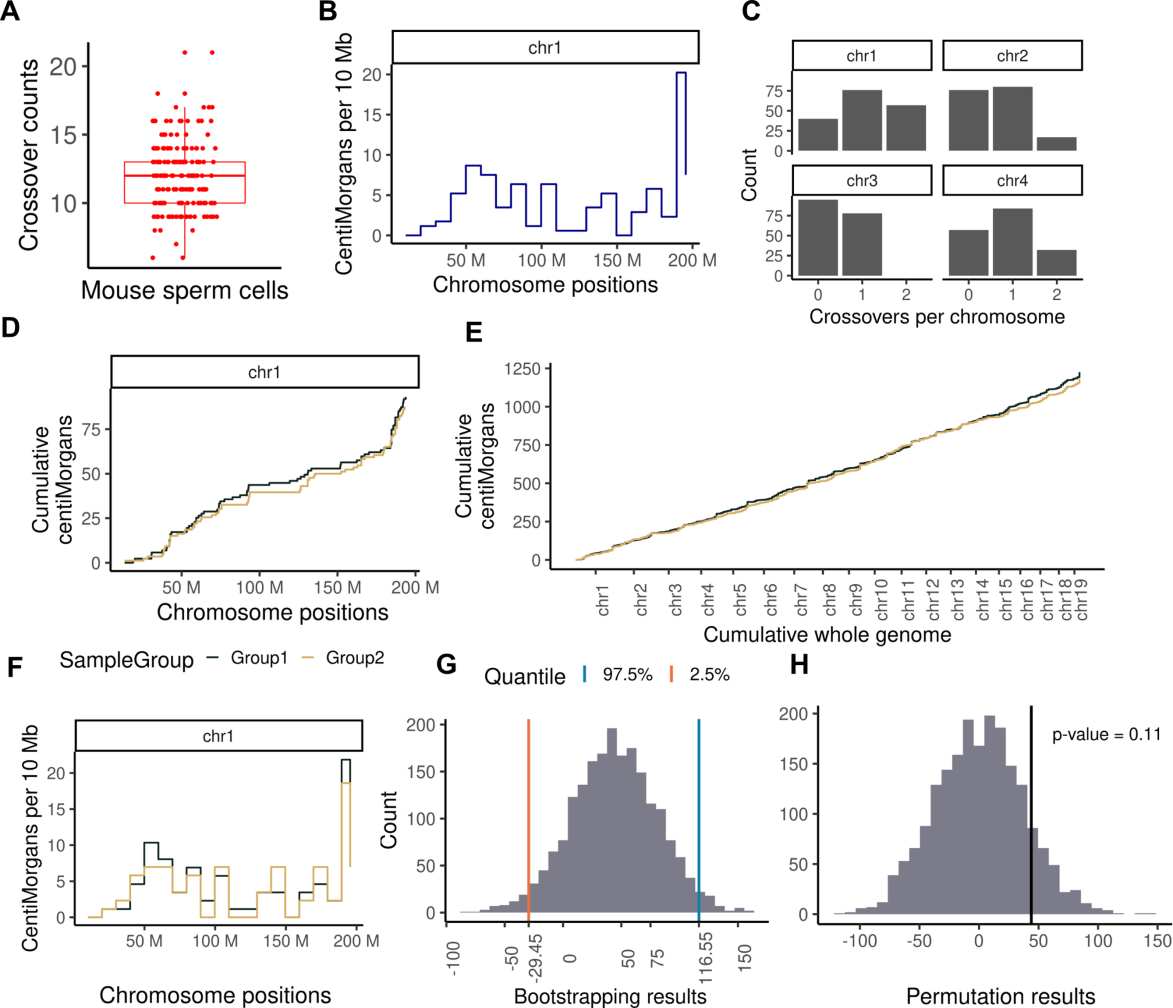
Application of sgcocaller and comapr on mouse sperm dataset (21). comapr enables easy plotting of crossover summary statistics. (**A**) The distribution of crossover counts per sperm (*n* = 173 sperm cells). (**B**) The genetic distances (in centiMorgans, which were derived from observed crossover rates by applying the mapping function Kosambi (42) with implemented function in comapr) plotted for every 10 megabase bin along chromosome 1. It has shown a low to zero crossover rate around the centromere region (125M). (**C**) The frequency of crossover counts for four chromosome are plotted for all sperm cells. (D, E) The cumulative genetic distances are plotted for chromosome 1 and cumulatively whole genome for the two randomly assigned mouse sperm groups. (**F**) The binned (10 Mb) genetic distances plot along chromosome 1 for the two sperm groups. (**G**) The bootstrapping distribution of the difference in total genetic distance between the two groups with two vertical lines indicating the lower bound and the upper bound of 95% confidence interval. (**H**) Permutation results of difference in total genetic distance between two sperm cell groups with observed group difference indicated by the vertical line and p-value labelled on the top.

We applied sgcocaller and comapr to haplotype sperm cells from a published DNA sequencing dataset of individual mouse sperm cells collected from an F1 hybrid mouse (C57BL/6J X CAST/EiJ) (21). The raw sequencing dataset was downloaded from GEO (Gene Expression Omnibus) with accession GSE125326 (see Supplementary Methods) and the workflow with detailed steps for pre-processing the raw reads and the execution of *sgcocaller|comapr* is publicly available (see Data Availability). We demonstrate and provide reproducible code for running the functions to generate plots of the number of crossovers per sperm cell (Figure 4A), genetic distances in the chosen size of chromosome bins along chromosomes (Figure 4B), and the number of crossovers (COs) identified per chromosome (Figure 4C) in the public repository (see Data Availability).

To demonstrate the functions in comapr that test for differences in groups of cells, such as cells from different individuals, we divided the sperm cells into two groups randomly. The crossover counts and crossover rates of each group are calculated over the SNP intervals via function countCOs and genetic distances are derived by the calGeneticDist function that applies a user-selected mapping function such as the Kosambi mapping function (42). Comparative plots can be generated for the two groups including genetic distance plots for binned intervals along the chromosome and the cumulative genetic distances along a chromosome or whole genome (Figure 4D–F). Resampling-based testing functions, bootstrapping and permutation, have been called to assess the group differences statistically (Figure 4G, H). The bootstrapping testing was conducted via bootstrapDist function. The bootstrap 95% confidence interval of the group differences was calculated. The interval included zero, indicating that there was not enough evidence to support a difference in mean crossover numbers between the two groups at a significance level of 0.05, which was expected because the group labels of sperm cells were randomly assigned (Figure 4G). The permutation testing via permuteDist returned the same conclusion with a permutation *P*-value >0.05 (Figure 4G, H and see Materials and Methods).

**Figure 5.**
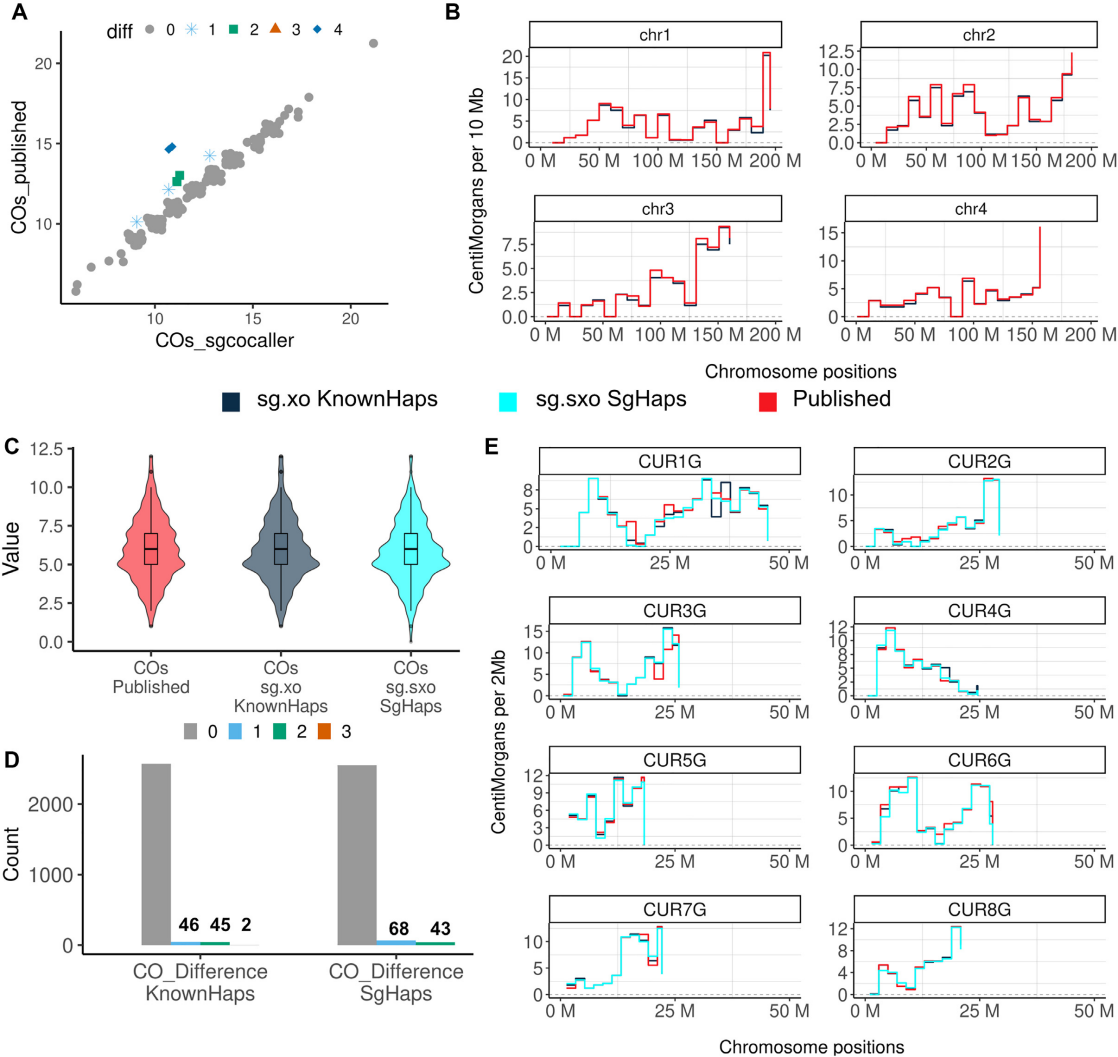
Comparisons between number of crossovers called from sgcocaller versus from the published study. (**A**) The number of crossovers (COs) called per sperm by sgcocaller (x-axis) versus by the original paper (y-axis) are plotted; points were jittered and colored and shaped by the differences in number of crossovers by the two methods. (**B**) The genetic distances in 10 Mb chromosome bins were calculated and plotted along the chromosomes from using either crossover rates estimated from *sgcocaller xo* with the known haplotypes or the obtained published results of the mouse sperm dataset (21). (**C**) The distribution of the number of crossovers called per apricot gamete (*n* = 333 apricot gametes) from the published study (20) (COs Published), calling *sgcocaller xo* using the published haplotype (sg.xo KnownHaps) and calling *sgcocaller sxo* with *sgcocaller phase* generated haplotype (sg.sxo SgHaps). (**D**) The frequency of the difference in crossover counts with published crossover results per chromosome by sgcocaller using either the published haplotype (CO_Difference KnownHaps) or using *sgcocaller phase* generated haplotype (CO_Difference SgHaps). (**E**) The genetic distances in every 2 Mb chromosome bin were calculated using crossover rates estimated from three approaches that showed high concordance along regions of the genome.

The crossovers called by *sgcocaller|comapr* (with a mean number of crossovers of 12 per sperm across autosomes) were highly consistent with the crossovers called from the original paper. There were 7 cells (4%) that were called with a different number of crossovers compared to the original paper (Figure 5A). We also compared the genetic distances calculated when using the estimated crossover rates by *sgcocaller xo* and the published study (21) in 10 Mb intervals, and it showed perfect alignment between the two methods (Figure 5B and Supplementary Figure S3).

#### 10X scCNV apricot gametes

We next applied sgcocaller and comapr on the 10X scCNV apricot pollen dataset (20) after pre-processing (see Materials and Methods; Supplementary Methods) for crossover calling and compared the called crossovers by our toolset with the published results.

Comparing with the crossover profile constructed from the original study, sgcocaller and comapr have generated a highly concordant crossover profile for the analysed gametes (Figure 5C–E, Figures S3 and S4). The number of crossovers identified per gamete (*n* = 333 gametes) was consistent with the published number of crossovers for these gametes by using both the published haplotype (sg.xo KnownHaps) and the *sgcocaller phase| swphase* generated haplotype (sg.sxo SgHaps) (Figure 5C). Few chromosomes (2% by sg.xo KnownHaps; 3% by sg.sxo SgHaps) of the 2664 chromosomes studied here exhibited differences in crossover counts (Figure 5D). Out of the chromosomes that were called with different numbers of crossovers, 56% of them were called with fewer crossovers by sg.xo KnownHaps and 68% with fewer crossovers by sg.sxo SgHaps. Therefore, the discrepancy of results from the two methods resulted predominantly from sgcocaller being more conservative and calling fewer crossovers. Inspecting the crossover profile by chromosome regions has also demonstrated consistent genetic distances across the whole genome (Figure 5E).

It is worth noting that not all cells available in the published study were analysed and included in the application results of sgcocaller due to the cell filtering step included in comapr which filtered out cells with excessive numbers of crossovers called and cells with poor SNP coverage. Chromosomes with excessive numbers of crossovers called are likely due to library preparation artefacts or abnormal chromosome segregation that results in implausibly many heterozygous SNPs in single gametes (Supplementary Figure S5). comapr offers a systematic way of filtering out these cases and can be applied to all gametes. We provide guidelines in false crossover filtering from analysing different datasets (see Supplementary Methods and Supplementary Figure S6).

### Simulated datasets with increased crossovers

To test the performance of calling crossovers from gametes with increased crossovers comparing to the apricot and mouse sperm gametes, we simulated 100 cells each with 6 manually inserted crossovers spanning a 5M genomic region (see Supplementary Methods). We phased the hetSNPs in the 5M region and called crossovers for the 100 cells using sgcocaller. Only 95 hetSNPs (out of 21k hetSNPs) were phased incorrectly and all six crossovers were discovered for the 100 cells (Supplementary Figure S7).

### sgcocaller advances performance and efficiency

**Figure 6.**
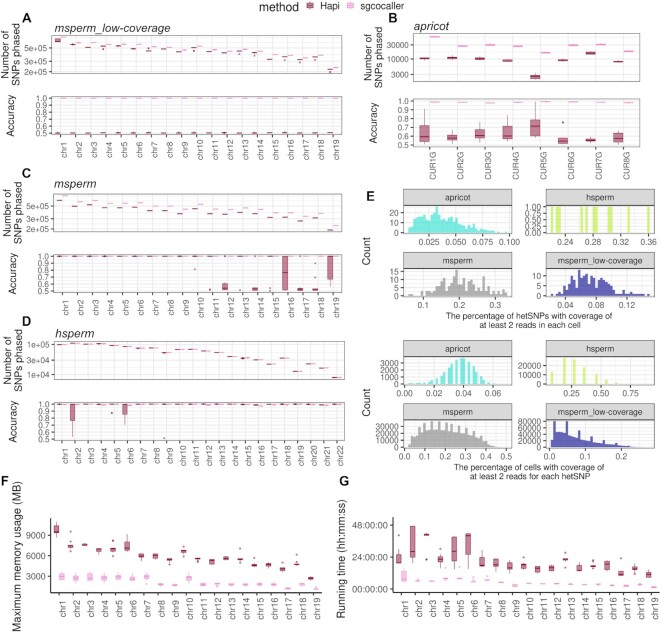
Phasing module performance comparison with existing method. The phasing performance of sgcocaller and Hapi were compared on the constructed dataset repeats from four different datasets, msperm_low-coverage, apricot (20), msperm (21), and hsperm (28) (Supplementary Table S1). (**A**) The number of phased SNPs and phasing accuracy from running two methods (sgcocaller and Hapi) on 10 constructed datasets from using the msperm_low-coverage sperm cells were compared (10 dataset repeats, each with 174 or 175 cells). msperm_low-coverage dataset was generated by downsampling DNA reads from msperm dataset. (**B**) Same as (A) but for the constructed 10 apricot gamete datasets (each dataset with *n* = 330 or 331 gametes). Hapi failed when executing for two chromosomes and did not return values (2 out of 10 × 8 = 80 in total), which were not included in the plot. (**C**, **D**) The number of phased SNPs and phasing accuracy from running two methods on the 11 constructed datasets using 11 human sperm cells (hsperm) , and on the 10 constructed datasets using mouse sperm cells were plotted in boxplots. For human sperm dataset, Hapi failed to return values for four chromosomes (out of 11 × 22 = 242 in total), which were not included in the plots. (**E**) The characteristics of the four datasets were compared by comparing the SNP coverage rate and cell coverage rate using the first chromosome in each dataset. (**F**, **G**) The running time and memory usage by the two methods for phasing each chromosome using the constructed 10 datasets from the mouse sperm dataset (21) were compared. Time was reported in format of hour:minute:seconds, and memory was measured in units of mega bytes (MB). Measurements were reported by the ‘benchmark’ function from Snakemake (47).

Many studies apply hidden Markov model based approaches for haplotype construction and crossover identification using sequencing or genotyping datasets (9,21,43–46). The majority of them, however, used customised or in-house scripts for crossover calling (9,21,43). Only a handful of them published reusable software tools or pipelines (39,45), but none of them apply in the same scenarios as sgcocaller and comapr, which work directly on large-scale single-cell DNA sequencing datasets. The Hapi method, implemented in an R package, was proposed to construct chromosome-scale haplotypes using data from a small number of single gametes (39). Given a genotype matrix as input, it has functionalities overlapping with *sgcocaller phase* (39). We therefore compared the phasing performance of Hapi and *sgcocaller phase|swphase* on a total of four datasets: the individually sequenced mouse sperm cells (21), the 11 human sperm cells (the same dataset used in the performance evaluation of Hapi in the original paper (28,39), the apricot gamete dataset generated by the 10X scCNV protocol (20), and lastly the low-coverage mouse sperm dataset that is more comparable to a contemporary droplet-based single-gamete DNA sequencing dataset (Figure 6E) (see Supplementary Methods). To measure uncertainty in method performance, we constructed dataset replicates by dividing the original sets of gametes into ten portions and each time leaving out one portion (10%) of the gametes. Since there were only 11 cells in total in the human sperm cell dataset, 11 dataset replicates were constructed (n = 10 cells each). Ten distinct datasets were constructed from the apricot gametes (*n* = 330 or 331 gametes each), mouse sperm dataset (*n* = 174 or 175 sperm cells), and the low coverage mouse sperm dataset (*n* = 174 or 175 sperm cells). We ran Hapi and *sgcocaller phase|swphase* on these constructed datasets. The phasing accuracy, measured by calculating the fraction of SNPs agreeing with the ground-truth haplotype sequence, and the number of phased SNPs by each approach were compared (Figure 6A–D).

On the lower coverage datasets, (the apricot gamete dataset and the low-coverage mouse sperm dataset), the advantage of *sgcocaller phase|swphase* is readily apparent with far more SNPs phased and much higher accuracy. *sgcocaller phase|swphase* generated haplotypes that were concordant with the published haploid genome assembly with lowest accuracy across all chromosomes in all dataset repeats of 97.6% for the apricot dataset. All chromosomes were phased with accuracy above 99.99% for the low-coverage mouse sperm datasets by *sgcocaller phase|swphase* (Figure 6A, B). We also validated that *autophase*, which runs *sgcocaller swphase* automatically after sgcocaller phase, can produce the same output as *sgcocaller phase|swphase* (Supplementary Figure S8). In contrast, Hapi delivered median accuracy below 75% for the apricot datasets and only 50% accuracy (equivalent to random guessing) for the low-coverage mouse sperm datasets. Hapi’s poor accuracy on these datasets makes its results unusable for downstream applications like crossover calling.

On the high coverage datasets (the mouse sperm dataset and the human sperm dataset), *sgcocaller phase|swphase* achieved equivalent or greater haplotype completeness in terms of the number of phased SNPs compared to Hapi (Figure 6C, D). *sgcocaller phase|swphase* achieved near perfect accuracy for all sperm combinations (all above 0.99), whereas Hapi repeatedly generated unusable phasing results (accuracy <0.6) for chromosome 12, 14, 17, 18 for the mouse sperm datasets (Figure 6C). Hapi did not perform stably for the 11 human sperm datasets, but *sgcocaller phase|swphase* always generated phasing results with accuracy >97% across all chromosomes in all human sperm combinations. (Figure 6D). Hapi also failed for four chromosome runs that triggered errors (out of 242 chromosomes tested across all datasets), which were excluded from the comparison.

We compared the differences among the four datasets, which showed that the human sperm sequencing dataset and the mouse sperm sequencing data have much higher SNP coverage per cell and cell coverage per SNP compared to the apricot dataset (Figure 6E). The low coverage mouse sperm dataset has coverage distributions that are more comparable to the apricot dataset.

In summary, *sgcocaller phase|swphase* outperforms Hapi on both high coverage data and low coverage data with *sgcocaller phase|swphase* more dominant in the low coverage datasets.

#### Computational efficiency

We compared the computational efficiency of the two methods (sgcocaller and Hapi) on the large mouse sperm dataset and measured the running time as well as memory usage on each of the ten dataset repeats. The running time required by *sgcocaller phase* plus *sgcocaller swphase* was only 11–38% of the total time taken by Hapi (comparing the median running time of each chromosome). Considering *sgcocaller phase* timing results includes an extra step of parsing the DNA read file (BAM file) and variant file (VCF file), *sgcocaller phase* is even more advantageous in terms of speed than these results indicate. Examining diagnostic plots generated from *sgcocaller phase* can help with identifying switch errors so that *sgcocaller swphase* can be run selectively. Nevertheless, *swphase* was applied to all chromosomes and the total running times were still many-fold shorter than Hapi. In terms of memory usage, since *sgcocaller phase* and *sgcocaller swphase* were run consecutively, the process that used larger memory was regarded as the memory used by sgcocaller. From comparing the reported max_rss (the maximum amount of memory occupied by a process at any time that is held in main memory (RAM)) by Snakemake (47), sgcocaller required only 30–50% of the memory required by Hapi (Figure 6F, G).

### Scalability of sgcocaller

We tested the scalability of different modules in sgcocaller with input files of varying sizes including different numbers of cells, numbers of DNA reads per cell, and numbers of hetSNPs available. We ran each module and recorded the computational usages by each module on the simulated datasets (See Supplementary Methods and Supplementary Figure S9A, B). Across all simulated datasets, all programs can be finished in 6 hours using less than 5GB of memory excluding *sgcocaller sxo*. We observed substantial improvement of processing speed of *sgcocaller sxo* over *sgcocaller xo* but *sgcocaller sxo* also required much more memory for larger datasets. Therefore, we would recommend using *sgcocaller xo* with the generated phased VCF from sgcocaller instead of running *sgcocaller sxo* when processing large datasets (when analysing >1500 cells with >400k SNPs) and memory is not sufficient.

To characterise the current scale of single-gamete sequencing datasets, we obtained the DNA sequences of sperm cells from donor 1 in a large-scale study (9). We selected the top 3000 cell barcodes with the largest number of DNA reads. We counted the number of hetSNPs and mean number of DNA reads per cell for each chromosome after pre-processing (see Supplementary Methods, and Supplementary Figure S9C). Lastly we applied sgcocaller phase, swphase and sxo on these sperm cells and measured the running times and memory usages of each program (Supplementary Figure S9D, E). Each program was executed with three repeats and the computational usages were recorded using the benchmark function in Snakemake(47). For all the chromosomes tested, each module was finished in under 2 hours with maximum memory usage under 2.5 Gb (Supplementary Figure S9D, E). In conclusion, sgcocaller can handle current and larger-scale single-gamete datasets.

## DISCUSSION

We have introduced a toolkit that consists of a command-line tool and a Bioconductor/R package for processing large-scale single-gamete DNA read datasets for individualised haplotype construction, crossover identification, and crossover landscape analysis. Personal haplotype construction is important in population genetics and clinical genetics for interpreting rare and disease-implicated variants (48,49). Genomes of haploid gametes provide information of ‘long-range’ haplotype blocks of the diploid donors and can be revealed via standard short-read sequencing. Single-gamete data generated using low-depth short-read sequencing are sufficient for reconstructing the two personal haplotypes by aggregating linkage information in a group of gametes.

Existing read-based haplotype construction methods have also been applied on single-gamete sequencing datasets. For example, HapCUT2 (26) was applied on human sperm datasets for generating individualised haplotypes (9). However, applying HapCUT2 to single-gamete datasets requires extra pre-processing of the aligned DNA reads from gametes for generating the single gamete genotypes, from which a specially formatted fragment file can be constructed (see Supplementary Methods). However, sgcocaller reduces these extensive processing steps and wraps them into one (two) module(s) specifically designed for phasing single-gamete datasets with better phasing performance than HapCUT2 (Supplementary Figure S10).

Taking advantage of SNP linkage information in single-gamete data, *sgcocaller phase* is able to construct personal haplotypes with standard short-read sequencing methods to near perfect accuracy. Comparisons with other phasing methods demonstrate that *sgcocaller phase* offers better performance on single-cell sequenced gametes in terms of accuracy and efficiency. Crucially, haplotypes constructed with *sgcocaller phase* solely from single-gamete sequencing data are sufficiently accurate to support highly accurate downstream crossover calling with sgcocaller’s crossover calling methods. sgcocaller achieves highly accurate phasing results in settings where competitors fail to generate usable haplotypes for crossover calling. The outputs of sgcocaller can be conveniently imported into R with the comapr package for sophisticated crossover landscape visualisation and analysis.

The list of hetSNPs can be identified from the pooled DNA reads from all gametes as we demonstrated. The list of hetSNPs can also come from other sources such as whole genome DNA sequencing of the sample. Applying stringent filtering is helpful for obtaining the list of higher quality hetSNP markers. For example, filtering of hetSNPs to remove SNPs in genome regions that may cause problems in alignment and genotyping (e.g. centromeres, highly repetitive regions, paralogous regions) is also beneficial when running sgcocaller phase and crossover calling.

Our gamete-based haplotype phasing model has limitations. The model uses the hetSNP linkage information in the gametes and assumes the nearby hetSNPs are close and crossovers rarely break the linkages among them. For cases where the density of hetSNPs is low, but evenly distributed along the chromosome region, the phasing model can still produce correctly phased haplotypes with reduced completeness (the number of phased hetSNPs is low; Supplementary Figure S11C). However, it is more problematic when phasing individuals which have large regions of homozygosity, e.g., F1 samples from two closely related parental strains. The hetSNPs located near the large regions of homozygosity may have been assorted to gametes independently and may presumably violate the linkage assumption. Thus, the phasing model may fail and could potentially generate phased haplotypes with switch errors. Since the power to detect crossovers is closely related to the number of hetSNPs, access only to sparse hetSNPs reduces the ability to detect crossovers and the resolution of the crossovers detected (Supplementary Figure S11A, B).

Due to the current design of the methods, *sgcocaller|comapr* are limited to the analysis of gametes from diploid organisms. Currently, sgcocaller only supports using multiple threads for decompressing the input BAM file and recommends users to run each chromosome in parallel to reduce computational time. Future work in the development of sgcocaller might include updating the program to include the multi-threading feature by chromosomes internally and supporting analyses for polyploid organisms.

The application demonstrations of *sgcocaller|comapr* on public datasets show that *sgcocaller|comapr* are able to produce stable and higher accuracy results than other methods with greater convenience and computational efficiency for large datasets. Although *sgcocaller|comapr* are optimised to work with cell-barcoded DNA reads, we also demonstrated and showed examples of how our tools can be applied on bulk-sequenced individual gametes. Using the modern programming language Nim and building on top of C-based libraries, sgcocaller processes the large genomic datasets efficiently. The multiple release formats of sgcocaller enable it to be more accessible to the community.

We demonstrated the application of our models to datasets generated using the 10X single-cell CNV protocols. Datasets generated using protocols such as single-cell ATAC-seq offer a promising alternative approach and we predict that our models will work well on such datasets. However, different technologies generate datasets with different genome coverages and single-cell ATAC-seq datasets are likely to have less genome coverage than datasets produced with the single-cell CNV method. Therefore, the number of crossovers detected from single-cell ATAC-seq datasets is likely to be slightly lower. We are less optimistic on single sperm datasets generated using 10X single-cell RNA-seq methods for crossover identification due to the possible transcript sharing among gametes via cytoplasmic bridges (50). Although the sharing of transcripts may be biased towards the underlying haplotypes of the gamete, which may be used for inferring haplotypes of the gametes’ genomes for crossover identification (50), the low depth of coverage in 10X single-cell RNA-seq data makes it challenging. In settings that are not subject to transcript sharing among individual gametes, we expect our methods to perform well on single-cell RNA-seq data.

Advances in single-cell DNA sequencing technologies that generate larger-scale and higher-quality datasets enable exciting opportunities for research using personalised meiotic crossover landscapes (11). Abnormal meiotic crossovers are often associated with infertility (5). Therefore, characterising individual meiotic crossover profiles and studying crossovers as a phenotype using single-cell techniques has application in reproduction clinics. Fundamental mechanisms and factors affecting meiotic crossovers remain active research topics. Using single-gamete methods for generating and comparing crossover profiles of individuals with different conditions has many advantages over traditional approaches that require recruiting large sample sizes (11). Our software serves as a complete toolkit for the first step of studies to inspect the single gamete data of an individual for understanding individual-level variations in meiotic crossovers.

## CONCLUSION

Available in multiple release formats (nimble package, static binary, docker image, bioconda package), sgcocaller fills the gap of a highly accurate and efficient tool with simple installation for constructing personalised haplotypes and calling crossovers in single gametes from single-cell and bulk DNA sequenced gametes. The availability of the companion Bioconductor/R package comapr enables the downstream statistical analysis of crossovers in the cells and among individuals, integrating well with existing R packages for various visualisation and exploratory analysis tasks. In concert, these packages represent a comprehensive, user-friendly toolkit for the construction and analysis of personalised crossover landscapes from single-gamete sequencing data.

## DATA AVAILABILITY

Source code of the latest version of sgcocaller is publicly available at a GitLab repository and comapr at a GitHub repository under a MIT license. comapr is also available as a Bioconductor package. The datasets used in this study are public datasets with raw sequencing data downloaded from GEO with accession GSE125326 and ENA with project ID PRJEB37669. The analysis steps including pre-processing and scripts for generating the figures are openly accessible at GitLab repositories and workflowr (51) web pages demonstrating the full workflow of applying *sgcocaller|comapr* are available. The source code and analysis repositories included in this study are listed below:

- sgcocaller: https://gitlab.svi.edu.au/biocellgen-public/sgcocaller
- comapr: https://github.com/ruqianl/comapr and https://bioconductor.org/packages/comapr
- mouse sperm data analysis: https://gitlab.svi.edu.au/biocellgen-public/hinch-single-sperm-DNA-seq-processing
- apricot gamete analysis: https://gitlab.svi.edu.au/biocellgen-public/calling-crossover-from-single-gamete-sequencing-of-apricot
- appendCB: https://github.com/ruqianl/appendCB
- distributions: https://github.com/ruqianl/distributions
- web page report of analyses on msperm dataset: https://biocellgen-public.svi.edu.au/hinch-single-sperm-DNA-seq-processing/Crossover-identification-with-sscocaller-and-comapr.html

## Supplementary Material

gkac764_Supplemental_File
