# Supplementary material for: sgcocaller and comapr: personalised haplotype assembly and comparative crossover map analysis using single-gamete sequencing data
Source: Nucleic Acids Res. 2022 Sep 15;50(20):e118. doi: 10.1093/nar/gkac764 (PMC9723612; doi:10.1093/nar/gkac764)
Supplement: gkac764_Supplemental_File [file gkac764_supplemental_file.pdf]

Supplementary Material
sgcocaller and comapr: personalised haplotype assembly and comparative crossover map analysis using single-gamete sequencing data

Ruqian Lyu [1,2], Vanessa Tsui [3,4], Wayne Crismani [3,4], Ruijie Liu [1], Heejung Shim [2,*], and Davis J. McCarthy [1,2*]

July 23, 2022

[1]Bioinformatics and Cellular Genomics, St Vincent's Institute of Medical Research, 9 Princes Street, Fitzroy Victoria, 3065, Australia

[2]Melbourne Integrative Genomics/School of Mathematics and Statistics, Faculty of Science, The University of Melbourne, Building 184, Royal Parade, Parkville, Victoria,3010, Australia

[3]DNA Repair and Recombination Laboratory, St Vincent's Institute of Medical Research, 9 Princes Street, Fitzroy Victoria, 3065 Australia

[4]The Faculty of Medicine, Dentistry and Health Science, The University of Melbourne, Victoria 3010 Australia

Contents

1 sgcocaller phase 3
- 1.1 Single gamete genotype matrix 3
- 1.2 Template cell identification 3
- 1.3 Infer missing SNPs in the haplotype template 4

2 sgcocaller swphase 5
- 2.1 Switch score calculation 5

3 sgcocaller xo and sgcocaller sxo 7
- 3.1 The Hidden Markov Model 7
- 3.2 Measuring support for detected crossovers 8
  - 3.2.1 logllRatio calculation 8
- 3.3 The outputs 9

4 False crossover filtering in comapr 9

5 comapr - resampling-based methods 10

6 Phasing 10X scCNV apricot gametes 11

*H.S. and D.J.M. supervised this work. To whom correspondence should be addressed. Tel: + 61 3 9231 2480; Fax: + 61 3 9416 2676; Email:dmccarthy@svi.edu.au.

7 Low phasing accuracy bins in apricot dataset **11**

8 Calling crossovers **12**
8.1 Mouse sperm dataset 12
8.2 10X scCNV apricot data with known haplotype 12
8.3 10X scCNV apricot data with sgcocaller phased haplotype 12

9 Phasing performance comparison **13**
9.1 Human sperm cell dataset 13
9.2 10X scCNV apricot gametes 13
9.3 Mouse sperm dataset 13
9.4 Mouse sperm low coverage dataset 13

10 Applying HapCUT2 on the apricot gametes **14**

11 Scalability testing **14**
11.1 Dataset construction for scalability testing 14
11.2 Pre-processing human sperm cells from donor1 for scalability testing 14
11.3 Running sgcocaller on datasets for scalability testing 15
11.3.1 Simulated large scalability testing datasets 15
11.3.2 Human sperm cells from donor 1 15

12 Simulated sperm cells with increased crossovers **15**

13 Effect of low hetSNP density **16**

14 Supplementary figures **18**

1 sgcocaller phase

Haploid genomes of the pool of gametes collected from an individual are the results of meiosis and meiotic crossovers. Based on the known mechanisms of meiosis and crossovers, with respect to each crossover, half of the daughter chromosomes will inherit the crossover and half will not. For each chromosome, we aim to identify a template cell (gamete) that does not inherit crossovers, thus their haploid genotype sequences represent the original (maternal/paternal) haplotypes of the diploid donor (see Template cell identification). Depending on the expected number of crossovers per chromosome in the meiotic crossover process, the proportion of gametes that inherit no crossovers with respect to each chromosome is different. It is possible that every gamete has one or more crossovers inherited and the template gamete chosen hence has crossovers which introduce "switch errors" in the inferred haplotypes and they can be corrected by *swphase* (see Switch score calculation). Generally, though, crossovers are low frequency events across chromosomes and crossover positions are sparse. The SNP linkages in small chromosome regions across all haploid gametes are therefore reliable for reconstructing the donor's haplotypes.

1.1 Single gamete genotype matrix

To generate the two haplotypes of each chromosome for the diploid donor from gametes, the first step is finding the list of unphased hetSNPs (heterozygous SNP loci that differ between the maternal and paternal homologous chromosomes) by standard variant calling tools such as `bcftools` using pooled DNA reads from gametes. Only biallelic SNPs are considered in this step, therefore finding one haplotype also implicitly resolves the second haplotype by switching all alleles to its alternative at each hetSNP. In other words, the two haplotypes are bitwise complementary to each other. With the hetSNPs known, the DNA reads from each gamete are parsed and summarised into a genotype matrix for each chromosome with values of 1 or 2 corresponding to matching with the REF allele and the ALT allele at each unphased hetSNP. ALT allele read frequency (AF) is used for genotyping each SNP in each gamete and AF ≤ 0.3 is genotyped as 1 (REF) while SNPs with AF ≥ 0.7 are genotyped as 2 (ALT). Filtering options are available for excluding low mapping/base quality reads, low coverage SNPs per cell with the options *–minCellDP* and *–minTotalDP*.

1.2 Template cell identification

An ideal template cell for phasing each chromosome is a cell without crossovers and having sufficient SNP coverage regarding this chromosome. To find a cell without crossovers, *sgcocaller phase* finds three pairs of gametes with lowest genotype dissimilarity. The dissimilarity of two genotype sequences is calculated by finding the proportion (p) of discordant SNPs between the two sequences and we define the dissimilarity value as $min(p, 1-p)$. *sgcocaller phase* includes *maxDissim* as a user supplied option that controls how similar ($1-$*maxDissim*) it requires for two cells to be considered as template cells. *sgcocaller phase* finds a maximum of three cell pairs as potential template cells. When multiple template cell pairs are available, the cell with the highest SNP coverage is chosen as the template cell. This approach is based on the idea that when two gamete

chromosomes have no crossovers, either their genotype sequences will be the same (or at least very similar if the same parental haplotypes have been inherited) or totally different (when different parental haplotypes are inherited by the two chromosomes). There are rare cases when two gametes have crossovers at exactly the same positions, which also leads to high genotype similarity in the gametes. Such cases can be revealed by diagnostic plots (Fig. 2c,d) with the simple R script provided and then corrected by *sgcocaller swphase*.

An ideal template cell can be found for gamete populations in which gametes with zero crossovers with respect to one chromosome are relatively high in proportion. However, for meiosis with high expected crossovers per chromosome (e.g., human eggs), an ideal template cell might not exist in the gamete population. That would mean there are no template cell pairs identified given a *maxDissim* threshold. For these cases, the cell whose total SNPs is the 85% quantile among all cells is selected as the template cell and switch error correction should be applied (see Switch score calculation). The template cell to use can also be chosen manually by the user via the *templateCell* option.

1.3 Infer missing SNPs in the haplotype template

Upon forming a haplotype template, *sgcocaller phase* increases the completeness of the haplotype template by inferring the genotype of the missing SNPs (that is, SNPs with no read coverage) from the template using other gametes in which the SNP is available (that is, has read coverage). A haplotype template represents two actual haplotype (allele) sequences (h, h') that are bitwise complementary with respect to the REF and ALT alleles defined for the hetSNPs used; one haplotype is thus derived by changing all alleles from the complementary haplotype to their alternatives. Intuitively, we want to use linkage information from other gametes to "fill in the gaps" in the template haplotype by finding gametes with coverage at the missing SNP site. Loosely, if a gamete with coverage at the missing SNP has the same haplotype as the template, then we assume that the template should have the same allele at the missing SNP as the gamete with coverage. If the gamete with coverage has the complementary haplotype, then the missing SNP in the template should have the alternative allele.

More precisely, to infer missing SNPs' genotypes in the haplotype template, we need to find the linkage type of the SNP to the haplotype template. Only two types of linkages are possible due to the existence of two possible alleles (e.g., 1 or 2) for a given SNP. The first linkage type ("type 1") refers to gametes with the template haplotype and allele 1 at the missing SNP and gametes with the haplotype complementary to the template haplotype and allele 2 at the missing SNP; that is, the missing SNP should have allele 1 in the template (type 1:$\{(1, h), (2, h')\}$). The second linkage type ("type 2") is the inverse, such that the template should have allele 2 at the missing SNP (type 2:$\{(1, h'), (2, h)\}$). To determine the linkage type of the SNP in supporting gametes (that is, those with read coverage of the SNP), the nearby SNPs' genotype sequence in each supporting gamete (specifically, the 10 closest SNPs with read coverage in both the template gamete and supporting gamete) is first compared with the template haplotype at the matching positions to define the haplotype (template or complementary) of the supporting gamete. With the haplotype of the supporting gamete determined, the linkage type supported by the gamete immediately

follows. The posterior probabilities of linkage types of a missing SNP are calculated by looking at the number of gametes supporting each type of SNP linkage. Assuming a genotype error rate of 0.1 and that the two linkage types are equally likely to happen, the posterior probability of each linkage type of a missing SNP can be calculated:

t_1: {type 1 linkage counts} t_2: {type 2 linkage counts}

$$
\begin{aligned}
p(\text{type } 1|t_1, t_2) & \\
&= \frac{p(t_1,t_2|\text{type } 1)p(\text{type } 1)}{p(t_1,t_2|\text{type } 1)p(\text{type } 1)+p(t_1,t_2|\text{type } 2)p(\text{type } 2)} \\
&= \frac{p(t_1,t_2|\text{type } 1)}{p(t_1,t_2|\text{type } 1)+p(t_1,t_2|\text{type } 2)} \\
&= \frac{0.9^{t_1}0.1^{t_2}}{0.9^{t_1}0.1^{t_2}+0.1^{t_1}0.9^{t_2}} \\
p(\text{type } 2|t_1, t_2) &= \frac{0.9^{t_2}0.1^{t_1}}{0.9^{t_1}0.1^{t_2}+0.1^{t_1}0.9^{t_2}}.
\end{aligned}
$$

We use a threshold cut-off (default is 0.99 and it can be changed via the option $posteriorProbMin$) for determining whether a missing SNP can be inferred. Missing SNPs with posterior probabilities over the threshold are inferred to be the suggested linkage type. Applying this approach genome-wide, we can make maximal use of read coverage across all gametes to maximise the completeness of the template haplotype. After inferring missing SNPs from the template haplotype, the step of inferring SNPs is then performed against all hetSNPs in the template haplotype to correct any genotyping errors in the template haplotype.

2 sgcocaller swphase

When an ideal template cell is not used for phasing in the previous step, the chosen template cell may have crossovers leading to switching errors in the inferred haplotype (Fig. 2c). *sgcocaller swphase* is able to detect the switch errors and generate the corrected haplotype. To save unnecessary computing, *sgcocaller swphase* calculates the switch scores only for identified SNP bins whose positions have high risk of having switch errors. High risk SNP bins are found by firstly grouping all hetSNPs into bins of 2,000 consecutive SNPs with a moving step of 200 SNPs (both are changeable via options when running *sgcocaller swphase*). The proportion of gametes having crossovers are calculated for each bin. A SNP bin is labelled as a high risk bin when the proportion of gametes having crossovers is above 0.5. *sgcocaller swphase* calculates switch scores for SNPs positions potentially having switch errors. It is based on the idea that when the majority of gametes have crossovers according to the template haplotype, it indicates a “crossover” or switch error in the template haplotype. The crossover identification in this step is fast as it simply compares the dissimilarity of gametes’ genotype sequences with the inferred haplotype sequence for the SNPs in each bin. A default threshold value of 0.0099 is set for the dissimilarity to decide whether a crossover has happened in the gamete or not.

2.1 Switch score calculation

To construct the switch score (formed using a concept for splitting blocks similar to that from a previous haplotype construction method [1]), which represents

how likely a switch error has happened at SNP i in the inferred haplotype, the switched haplotype is first constructed. Let H_l^i represents the haplotype sequence to the left N bases of SNP i, and H_r^i represents the haplotype sequence to right N bases of SNP i. The current haplotype around SNP i is $H^i = \{H_l^i, H_r^i\}$. The switched haplotype is a new sequence of $H_{sw}^i = \{H_l^i, H_r^{i\prime}\}$, where $H_r^{i\prime}$ is the bitwise complementary sequence of H_r^i. The switch score for each SNP i is calculated using the log-ratio of the probability of observing the genotype sequences in all gametes given the switched haplotype with the probability of observing the genotype sequences in all gametes given the non-switched haplotype. Assuming the occurrence of a switch error or not at any site is random, that is the prior of having switch or no switch at a SNP site equals 0.5, the switch score is the log-ratio of the posterior probabilities of the switched haplotype and the not-switched haplotype. When calculating the probability of observing each gamete's genotype sequence given the haplotype (H^i or H_{sw}^i), only the local (N) SNPs are considered (controlled by the *lookBeyondSNPs* option with default set to 20).

The haplotype H^i is a sequence of alleles and also implicitly defines the second haplotype $H^{i\prime}$ which can be derived by switching all alleles to their complementary alleles (in the called genotypes of the hetSNPs). The probability of observing all gametes' genotype sequences at the local (N) SNPs around SNP i given the local haplotype $(H^i, H^{i\prime})$ is,

$$p(G^i \mid H^i, H^{i\prime}) = \prod_{j=1}^{m} p(G_j^i \mid H^i, H^{i\prime}),$$

where G^i represents local genotypes around SNP i of all (m) gametes, and G_j^i represents local genotypes at SNP i from gamete j. For each gamete j, the probability of its genotype sequence given $(H^i, H^{i\prime})$ is

$$p(G_j^i \mid H^i, H^{i\prime}) = \frac{p(G_j^i \mid H^i) + p(G_j^i \mid H^{i\prime})}{2}.$$

Similarly for the switched haplotype H_{sw}^i, the probability of observing the local genotypes of all gametes given

$$(H_{sw}^i, H_{sw}^{i\prime}),$$

$$p(G^i \mid H_{sw}^i, H_{sw}^{i\prime}) = \prod_{j=1}^{m} p(G_j^i \mid H_{sw}^i, H_{sw}^{i\prime}),$$

where

$$p(G_j^i \mid H_{sw}^i, H_{sw}^{i\prime}) = \frac{p(G_j^i \mid H_{sw}^i) + p(G_j^i \mid H_{sw}^{i\prime})}{2}.$$

In addition, probability of observing a gamete's genotype sequence given a haplotype sequence, $p(G_j \mid h)$, is calculated as (assuming genotype error rate 0.1 and using d as the number of different bases between the allele sequence in G_j and the allele sequence in haplotype allele sequence h):

$$p(G_j \mid h) = 0.1^d \times 0.9^{(K-d)},$$

where K is the number of co-existing SNPs in two allele sequences under comparisons. The switch score for SNP i is thus

$$S_i = \log \frac{p(G^i \mid H^i_{sw}, H^{i\prime}_{sw})}{p(G^i \mid H^i, H^{i\prime})}.$$

Upon calculating switch scores for a sequence of SNP positions, the switching point is identified as the peak of a stretch of positive switch scores (Fig. 2b). The minimum threshold for identifying switch points is set via the option *minSwitchScore*, which can vary depending on features of the dataset including the number of cells available. The template haplotype is then corrected by flipping all SNP alleles to their complementary alleles after an identified switch point, thus generating the corrected haplotype with the switch error removed.

sgcocaller autophase validation

To test if the module autophase generates the same phasing results as running sgcocaller phase and sgcocaller swphase separately. we re-anlyzed chromosome 3 using one dataset out of the ten constructed msperm-lowcovarege datasets. sgcocaller autophase was called with options (combined options from running sgcocaller phase, sgcaoller swphase before): "–minDP 2 –maxTotalDP 150 –maxDP 10 –minSNPdepth 1 –maxDissim 0.0099 –binSize 1000 –stepSize 800 –lookBeyondSnps 10".

3 sgcocaller xo and sgcocaller sxo

Crossovers can be detected by finding haplotype shifts in the gametes' haploid genomes. With DNA reads from each cell mapped, the haplotypes of SNP markers are inferred by looking at the alleles carried by the DNA reads mapped to genomic positions of these SNP markers. However, with technical artefacts (from sequencing and mapping), it is expected to observe some proportion of conflicting alleles from the underlying haplotype (Fig. 1b). To reconstruct the haplotype structure of the haploid genomes from the mapped DNA reads while accounting for technical noise including mapping errors for crossover identification, *sgcocaller* applies a Hidden Markov model (HMM) with a binomial emission model (Fig. 2e). Breifly, we use a two-state HMM with states representing the haplotype origins of DNA segments in the gametes' genomes, and use the abundance of haplotype alleles at each SNP for inferring state transitions. Transitioning from one state to another between two SNP markers corresponds to a crossover detected.

3.1 The Hidden Markov Model

The two hidden states in the HMM represent the haplotype origins of DNA segments (represented by allele types of a list of SNPs) in the gametes' genomes. State transitions correspond to detected crossovers. We set the transition probability be dependent on the two SNP markers' base pair distances (physical distances) [2]. The transition probability is programmed as an configurable option (*cmPmb*) in *sgcocaller xo*. The relationship between observed allele counts

and the underlying hidden states are modelled by the two binomial distributions whose success rates are also user-configurable options (*–thetaREF, –thetaALT*) in *sgcocaller xo* (Fig. 2e and MATERIALS AND METHODS). The two states (named as REF and ALT) match with the alleles (bases) in the REF and ALT fields in the input VCF. The binomial distributions in the HMM model the ALT allele read counts at each SNP site and thus the *–thetaREF* value is expected to be small (e.g., 0.1) while *–thetaALT* value is expected to be higher (e.g., 0.9). The data in this model are the allele-specific read counts across the list of hetSNP sites for each chromosome, whereas the underlying haplotype of a SNP site on the chromosome is a hidden variable to be inferred. In the case of gametes, which have haploid genomes, there are two possible hidden states for each SNP corresponding to the two haplotypes of the parent. The two possible alleles at each hetSNP site can be referred as REF or ALT arbitrarily. The REF or ALT state for each SNP also aligns with the same REF or ALT allele in the provided VCF input file. At each SNP site i, the two hidden states: $s^i = \text{alt}$ corresponds to ALT haplotype while $s^i = \text{ref}$ corresponds to REF haplotype. The emission probabilities are modelled by two binomial distributions:

$$c^i_{\text{total}} = c^i_{\text{alt}} + c^i_{\text{ref}} \ ,$$

$$c^i_{\text{alt}} | s^i = \text{alt} \sim \text{Bin}(c^i_{\text{total}}, p_{\text{alt}}) \ ,$$
$$c^i_{\text{alt}} | s^i = \text{ref} \sim \text{Bin}(c^i_{\text{total}}, p_{\text{ref}}) \ ,$$

where c^i_{alt} and c^i_{ref} denote the alternative allele read count and reference allele read count at SNP i, respectively, c^i_{total} denotes the total read count at SNP i, and p_{alt} and p_{ref} are configurable parameters when running *sgcocaller xo* and denotes the success rates in the two binomial distributions respectively. The transition probabilities (p^i_{trans}) are modelled dependent on markers' base pair distances [2] with the default of average 0.1 centiMorgan per 1Mb (1 million base pairs) which can be changed via the *cmPmb* option. Lastly, the initial probabilities for the two hidden states are both set to be 0.5, making the assumption that they are equally likely to happen.

3.2 Measuring support for detected crossovers

We use a quantitative measure, the log-likelihood ratio (logllRatio), for measuring the amount of support from data for detected crossovers. We define a (Viterbi) state segment as a consecutive list of SNPs with the same state. We also use the term "inferred state" to refer to the state inferred through applying the Viterbi algorithm, whereas we use the term "altered state" of a SNP to refer to the state obtained by altering its inferred state to its opposite. We measure the support in the data for the detected crossover using the log-likelihood ratio for the segment with the inferred state, relative to the altered state.

Specifically, the logllRatio is calculated by taking the log-likelihood of the data given the current inferred state minus the log-likelihood of the data given the altered state (Fig. 2f)

3.2.1 logllRatio calculation

For a state segment that spans SNP q to k, assuming the state segment has been inferred with state REF,

$$\text{Logll}_{\text{inferred}} = \log(t_l) + \sum_{i=q}^{k} \log f(c^i_{\text{alt}}; c^i_{\text{total}}, p_{\text{ref}}) + \log(t_r),$$

where t_l and t_r denote the transition probabilities from SNP $q-1$ to q and from SNP k to $k+1$, respectively, and f is the binomial probability mass function given by

$$f(x; c, p) = \binom{c}{x} x^p (c-x)^{1-p}.$$

The log-likelihood of the data given the altered state is thus

$$\text{Logll}_{\text{altered}} = \log(1-t_l) + \sum_{i=m}^{k} \log f(c^i_{\text{alt}}; c^i_{\text{total}}, p_{\text{alt}}) + \log(1-t_r).$$

The logllRatio is derived by

$$\text{Logll}_{\text{inferred}} - \text{Logll}_{\text{altered}}.$$

3.3 The outputs

Due to the sparsity of single-cell datasets, we have used sparse matrices in Matrix Market format as the output for *sgcocaller xo*, which saves disk space and enables efficient parsing in downstream analysis. *sgcocaller xo* generates sparse matrices of the allele counts, inferred haplotype states (output of the Viterbi algorithm) for each SNP across each cell. Columns of these matrices correspond to the list of single gamete cell barcodes and rows correspond to the list of het-SNPs. A supplementary text file (*viSegInfo.txt*), which contains the summary features of inferred Viterbi segments (a list of consecutive SNP markers with the same inferred haplotype state) (Fig. 2f), is also provided and can be used for convenient post-processing such as filtering of false positive crossovers. The haplotypes of each SNP are inferred using the Viterbi algorithm [3] therefore the states/segments are also referred to as the Viterbi state/segments. Features including starting SNP position, ending SNP position, the number of SNPs supporting the segment, and the log-likelihood ratio of the Viterbi segment are recorded for each segment in the text file (*viSegInfo.txt*).

4 False crossover filtering in comapr

The Viterbi state transitions in the inferred state sequence after running sgcocaller xo and sgcocaller sxo correspond to crossovers detected (Fig. 2f). However, they might correspond to false crossovers called, especially when the Viterbi state transitions are close together due to the crossover interference phenomenon in meiosis [4], which means two crossovers are more likely to space distantly than randomly on a chromosome. Therefore, compar implements a crossover filtering function to eliminate the false positive crossovers. A combination of three metrics can be used for filtering, namely, bpDist (the base pairs covered by the stretch of the segment), minSNP (the number of SNPs supporting the segment)

and the minlogllRatio (see Measuring support for detected crossovers). Segments that do not meet the requirements of these thresholds are removed hence the introduced crossovers by the segments are filtered out.

To understand the distribution of logllRatio and how it varies in different datasets, we analysed the relationship of logllRatio and the chromosomal positions of the segments in the msperm and apricot datasets (with known phased hetSNPs) (Fig. S6a,b).The value of logllRatio correlated with the number of SNPs supporting each segment (Fig. S6c,d,e). We observed that using threshold of nSNP $>$ 30 or (logllRatio $>$ (100 150) worked well for filtering false crossovers for the two datasets.

As suggested by the logllRatio versus midpoint of segments plot (Fig. S6 a,b), the logllRatio has a decreased tail at both chromosome ends. Thus, gating on logllRatio might result in underestimated crossovers at the ends of the chromosome however it is dependent on the SNP density at the chromosome ends. We recommend users to check distributions of summary statistics (`perCellQC` and `perSegChrQC`) and do exploratory analyses for deciding filtering thresholds.

5 comapr - resampling-based methods

To test for differences in the number of crossovers between any two groups of cells, re-sampling methods, permutation and bootstrapping tests [5, 6, 7], have been implemented in *comapr*. The two resampling-based functions in *comapr* are able to either generate the to generate the empirical p-value or find the confidence intervals for the estimate of the group differences.

The `permuteDist` function performs permutation:

1. Record the observed difference in total genetic distances between the two groups of cells, d_{obs}.

2. Take the group labels vector and permute the group labels by randomly assigning the labels across cells and calculate the new difference with the newly-generated permuted grouping.

3. Repeat step 2 for B times (e.g., $B = 1,000$).

4. Calculate the permutation p-value by using the `permp` function from the `statmod` package [8] that calculates the appropriate p-values for the permutation test when permutations are sampled with replacement, avoiding the common pitfalls of under-estimated p-values [5, 8].

The steps for generating the bootstrapping confidence intervals of genetic distance differences in two groups (A and B) of cells are implemented in the `bootstrapDist` function:

1. Randomly draw n cells with replacement from cells in group A where n is the group size of A and calculate the total genetic distance d_1 with the cells drawn.

2. Randomly draw m cells with replacement from cells in group B where m is the group size of B and calculate the total genetic distance with the cells drawn d_2.

3. Calculate difference in total genetic distances by $d_1 - d_2$.
4. Repeat step 1-3 for B times (e.g., $B = 1,000$).
5. Calculate bootstrap confidence intervals for the sampling distribution of the difference in genetic distance using the `quantile` functions at a desired level from R (R Core Team, 2021).

Whether or not the acquired confidence intervals contain zero can be use to decide whether or not the difference is statistically significant at a desired level.

6 Phasing 10X scCNV apricot gametes

To obtain the list of unphased hetSNPs for the apricot sample, the REF and ALT alleles were swapped for every other position in called hetSNPs from pre-processing the dataset. With the newly created list of unphased hetSNPs, and BAM file containing DNA reads from 367 gametes (See Pre-processing public datasets), *sgcocaller phase* and *swphase* were applied to generate the phased haplotypes for the apricot sample. *sgcocaller phase* was called with options `--minDP 2 --maxDP 10 --maxTotalDP 80 --minTotalDP 6 --minSNPdepth 1 --posteriorProbMin 0.99`. *sgcocaller swphase* was called with default options and all cells were used for calculating switch scores.

7 Low phasing accuracy bins in apricot dataset

We observed certain bins with relatively lower phasing accuracies for chromosomes in the apricot dataset. We took two chromosomes, CUR1G and CUR3G, for further investigation. We first located the SNP bins with phasing accuracies lower than 0.8 in the two chromosomes, and we found 3 bins from chromosome CUR1G (Fig. S1a) and 6 bins from CUR3G (Fig. S1c). We obtained the genotype of these SNPs in all single apricot gametes and checked the rate of discrepancy of the genotype sequences (panel ii, Fig. S2) with the generated haplotype by sgcocaller or the published haplotype. The percentage of haplotype contradictory genotypes in each bin per gamete were calculated. Precisely, the genotype sequences for SNPs in each bin in each gamete were compared with the haplotype sequence (a column out of the two columns in i or in iii, Fig. S2), and the rate of discrepancy was calculated for all gametes (Fig. S1, a,c). Without losing generality, we always used the haplotype sequence (the column out of the two sequences in i or in iii, Fig. S2) that resulted in a smaller value of discrepancy for each gamete. We observed that using the haplotypes by sgcocaller resulted in more gametes having a discrepancy rate of zero. This result suggests that the haplotypes generated by sgcocaller matched the genotypes in the gametes better than the published haplotypes. For comparison, we also plotted the proportion of SNPs with discrepant genotypes for SNP bins with higher phasing accuracies (>=0.99) (Fig. S1b,d) and the numbers of gametes with rate of discrepancy as zero for SNPs in these bins were similar using either the haplotype by sgcocaller or the published haplotype.

8 Calling crossovers

8.1 Mouse sperm dataset

Crossover results for mouse sperm data were obtained from calling *sgcocaller xo* on the prepared phased hetSNPs and BAM file for 194 sperm cells as described before (see MATERIALS AND METHODS). The detailed code available in a public GitLab repository (see AVAILABILITY OF DATA AND MATERIALS). with following filtering settings. sgcocaller xo was called with options: `--maxTotalDP 450 --maxDP 10 --thetaREF 0.1 --thetaALT 0.9 --cmPmb 0.1`. The final crossover intervals were identified using *comapr* with detailed code available in a public GitLab repository (see AVAILABILITY OF DATA AND MATERIALS) with following filtering settings. Cells with chromosomes that had fewer than 200 SNPs available were filtered out. Cells with chromosomes that were called with more than 55 crossovers (before false crossover filtering) were removed because excessive number of crossovers indicates abnormal chromosomes (Fig. S5). False crossovers were filtered by removing crossovers that were induced by segments that were supported by fewer than 30 SNPs. We observed gating by the number of supporting SNPs was sufficient in removing false crossovers. Other filtering cutoffs were also set although we did not observe they removed extra crossovers than just using minSNP = 30.

1. minSNP=30, the segment that results in one/two crossovers has to have more than 30 SNPs of support.
2. minlogllRatio=150, the segment that results in one/two crossovers has to have minimal logllRatio value of 150
3. bpDist=10^5, the segment that results in one/two crossovers has to have base pair distances larger than 10^5.

8.2 10X scCNV apricot data with known haplotype

sgcocaller xo was applied on 367 apricot gametes to identify crossovers using the hetSNPs called (see Section Pre-processing public datasets) with option: `--thetaREF 0.2 --thetaALT 0.8 --maxTotalDP 100 --maxDP 10 --minTotalDP 6 --minDP 1 --cmPmb 2`. The following filtering thresholds were applied when filtering false crossovers using functions in comapr: minSNP = 30, minlogllRatio=0, maxRawCO = 20, minCellSNP=100. `bpDist` was set to 10^6 for chromosome 1 and scaled by relative chromosome size for the rest of the chromosomes.

8.3 10X scCNV apricot data with sgcocaller phased haplotype

sgcocaller xo was applied on *sgcocaller* phased haplotypes. The following options were applied `--minTotalDP 6 --minDP 1 --thetaREF 0.2 --thetaALT 0.8 --cmPmb 2 --maxDP 10 --maxTotalDP 100`. The following filtering thresholds were applied when filtering false crossovers using functions in comapr: minSNP = 10, minCellSNP = 100, maxRawCO = 10, minlogllRatio = 0. `bpDist` was set to 10^6 for chromosome 1 and scaled by relative chromosome size for the rest of the chromosomes.

9 Phasing performance comparison

The detailed function calls for applying the two methods on the constructed datasets using human sperm, apricot gametes and mouse sperm datasets are described below.

9.1 Human sperm cell dataset

Hapi was run following the tutorial example and the allowNA parameter in imputing missing genotypes function was set to 3. *sgcocaller phase* was run using options: `--threads 4 --barcodeTag CB --minDP 2 --maxDP 50 --maxTotalDP 200 --minTotalDP 6 --maxDissim 0.099--minSNPdepth 1 --maxExpand 1000 --posteriorProbMin 0.9` for each chromosome in each dataset. *sgcocaller swphase* was called for all chromosomes with `--lookBeyondSnps 150 --minSwitchScore 580` and `--minPositiveSwitchScores 100`.

9.2 10X scCNV apricot gametes

The haploid genome assembly "Currot" published previously was used as the haplotype ground truth. The alleles in the list of prepared hetSNPs (see Preprocessing public datasets) were swapped for every other position to create the list of unphased hetSNPs. Due to the sparsity of the dataset, the `allowNA` argument in the `hapiImupte` function of Hapi was raised to 310 to avoid triggering running errors. *sgcocaller phase* was applied with options `--threads 4 --minDP 2 --maxDP 10 --maxTotalDP 80 --minTotalDP 6 --minSNPdepth 1 --posteriorProbMin 0.99` and *sgcocaller swphase* was called with `--binSize 1000 --stepSize 800`.

9.3 Mouse sperm dataset

The alleles in the list of called hetSNPs (see Pre-processing public datasets) were swapped for every other position to create the list of unphased hetSNPs. The `allowNA` argument in the `hapiImupte` function of Hapi was set to 3 to avoid triggering running errors. *sgcocaller phase* was applied with options `--threads 4 --barcodeTag CB --minDP 2 --maxTotalDP 350 --maxDP 10 --minSNPdepth 5 --maxDissim 0.0099 --posteriorProbMin 0.99` and *sgcocaller swphase* was called with `--binSize 1000 --stepSize 800 --lookBeyondSnps 10`.

9.4 Mouse sperm low coverage dataset

For the further low coverage mouse sperm dataset, the allowNA was set to be 3 and *sgcocaller phase* was called with options `--threads 4 --barcodeTag CB --chrom wildcards.chr --minDP 2 --maxTotalDP 150 --maxDP 10 --minSNPdepth 1 --maxDissim 0.0099`. *sgcocaller swphase* was called with options `--binSize 1000 --stepSize 800 --lookBeyondSnps 10`.

10 Applying HapCUT2 on the apricot gametes

We use the same pre-processing steps as in [9] for generating the input fragment files that can be used as inputs for phasing haplotypes using the read-based haplotype phasing tool HapCUT2 [1]. Briefly, the mapped BAM file was processed to generate the genotype matrix file that contained the genotypes per cell regarding the list of unphased hetSNPs. The genotype matrix was then used for generating the fragment file by linking every two consecutive SNPs' genotypes in each cell into one fragment which was then supplied as input fragment file when running HapCUT2. HapCUT2 was run with default options. The phasing accuracies of HapCUT2 were calculated and compared with sgcocaller's results (Fig. S10), which demonstrated the advantageous phasing performance of sgcocaller over HapCUT2.

11 Scalability testing

11.1 Dataset construction for scalability testing

We constructed simulated sperm cells (with chromosome 1 only) by sampling DNA reads from sperms in the msperm dataset. Using different random seeds, 16 sampled cells were generated using DNA reads in each sperm in the msperm dataset with the selected subsampling rate (0.04 or 0.08) using `samtools-v1.10` [10]. Therefore, in total ($16 \times 194 = 3,104$) cells were generated under each subsampling rate. We then constructed datasets containing different numbers of cells ranging from 1,000 to 3,000 by merging randomly sampled cells from the generated 3,104 sperm cells under each subsampling rate.

11.2 Pre-processing human sperm cells from donor1 for scalability testing

To characterise the scale of single-gamete read data generated in a recent large-scale study Bell et al (*Nature*, 2020), we obtained raw sequencing reads of sperm cells from donor 1 downloaded from NCBI with accession codes: SRR10140439, SRR10140440, SRR101404, SRR10140461, SRR10140462. Following the analysis steps described in the original study, we used DropSeq tools (v2.4.0) `TagBamWithReadSequenceExtended` for generating the unaligned BAM file with cDNA reads tagged by cell barcode (XC) and sample name (XM). `FilterBam` from DropSeq tools (v2.4.0) was called to remove the reads tagged with XQ (low quality reads). The tagged cDNA reads were then converted to fastq files with tags XC and XM remained in the sequence names using `samtools-v1.10` [10]. The reads in the fastq files were then mapped to the human reference genome hg.19 using minimap2-v2.20 [11]. PCR duplicates in the mapped BAM file were identified and removed by `gatk4.2 MarkDuplicates`. Heterozygous SNPs were called for donor1 using `gatk4.2 HaplotypeCaller` and only SNPs were kept. The SNPs were filtered by `gatk4.2 VariantFiltration` with options " `--filter-expression DP` $< 2||$ `QD` $< 10.0||$ `FS` $>$ $60.0||$ `SOR` $> 3.0||$ `MQ` $< 30.0||$ `MQRankSum` $< -12.5||$ `ReadPosRankSum` < -8.0" and SNPs that were concordant with the SNPs in the downloaded `dbsnp_138.hg19.vcf.gz` from dbSNP database were kept [12]. We filtered

the cell barcodes by their number of DNA reads available and the top 3,000 cell barcodes were kept.

11.3 Running sgcocaller on datasets for scalability testing

11.3.1 Simulated large scalability testing datasets

We applied sgcocaller on the constructed datasets and the computational resources required were recorded for all datasets (Fig. S9a,b). In addition to the number of cells and the mean number of DNA reads per cell, we also varied the number of input hetSNPs (columns of Fig. S9a,b). Modules of sgcocaller were called on all the simulated datasets with the same options and listed below, and each module was run with three repeats:

- sgcocaller phase: "`--minDP 2 --maxDP 10 --minSNPdepth 20 --maxDissim 0.0099`"
- sgcocaller swphase "`--binSize 1000 --stepSize 500 --dissimThresh 0.1 --lookBeyondSnps 10 --maxUseNcells 200`"
- sgcocaller sxo "`--thetaREF 0.1 --thetaALT 0.9 --cmPmb 0.1 --chrom chr1 --notSortMtx --batchSize 3000`"
- sgcocaller xo "`--minDP 2 --maxDP 10 --minSNPdepth 20 --thetaREF 0.1 --thetaALT 0.9 --cmPmb 0.1`"

sgcocaller sxo was called with phased haplotypes from *sgcocaller swphase* and allele count matrices from *sgcocaller phase*, while *sgcocaller xo* was called with the BAM file and the list of known phased hetSNPs in a VCF file.

11.3.2 Human sperm cells from donor 1

With the de-duplicated (PCR duplicates removed) DNA reads from sperm cells from donor 1 in Bell et al (*Nature*, 2020), and the prepared list of hetSNPs (see Pre-processing human sperm cells from donor1), *sgcocaller phase* was first called with options: "`--minDP 1 --maxDP 20 --maxExpand 3000 --posteriorProbMin 0.9 --minTotalDP 10`". The phased haplotypes from sgcocaller phase were further processed by *sgcocaller swphase* to correct switch errors in the inferred haplotypes: "`--binSize 2000 --stepSize 200 --dissimThresh 0.05 --lookBeyondSnps 10 --maxUseNcells 500`". *sgcocaller sxo* was called subsequently for detecting crossovers in the cells with phased haplotypes by *swphase* with options: "`--thetaREF 0.2 --thetaALT 0.8 --cmPmb 1 --batchSize 3000 --notSortMtx`"

12 Simulated sperm cells with increased crossovers

To test how our software tool performs on gametes with more frequent crossovers than the apricot gametes, mouse, and human sperm cells, we generated simulated cells with crossovers manually inserted. We generated a dataset with 100 sperm cells (with DNA reads mapped to a 5M region, coordinates 50Mb to 55Mb on chromosome 1 only) each with 6 inserted crossovers to test the performance of sgcocaller on gametes generated from meiosis with more frequent crossovers.

To generate the 100 sperm cells dataset, we first identified sperm cells in msperm in which no crossovers were detected on chromosome 1, which means their chromosome 1 either had the reference haplotype or the alternative haplotype from the donor. We selected 5 reference haplotype sperm cells and 5 alternative haplotype sperm cells with respect to chromosome 1. We then pooled together the 5 reference sperm cells to generate the pool of DNA reads from only the reference haplotype, and took the same approach for the alternative haplotype sperm cells. To insert 6 crossovers in each simulated sperm cell, 6 break points were selected for each cell. To generate sperm cells with different crossover positions, we first created a list of potential break point positions (with a distance of 125k base pairs per break point, 39 break points in total) in the 5M chromosome region. We sampled randomly 6 break points for each cell as the inserted 6 crossovers. The 100 simulated cells were simulated by sampling DNA reads from the reference haplotype DNA reads or the alternative haplotype DNA reads alternatively in alternating chromosome regions divided by the break points. The mean number of DNA reads per cell was 62k. We ran *sgcocaller autophase* with "`--minDP 2 --maxTotalDP 350 --chrom chr1 --maxDP 10 --minSNPdepth 5 --maxDissim 0.0099 --binSize 1000 --stepSize 800 --lookBeyondSnps 10`" for generating the haplotypes (Fig. S7a). We applied sgcocaller sxo subsequently for detecting crossovers in the 100 cells with options "`--thetaREF 0.1 --thetaALT 0.9 --cmPmb 1 --chrom chr1 --batchSize 50`". We filtered out segments that were covered with fewer than 30 SNPs, and the final number of crossovers was counted for each cell by finding state transitions (Fig. S7b).

13 Effect of low hetSNP density

To demonstrate the effects of low SNP density, we took the simulated 100 sperm cells each with 6 crossovers in a 5M chromosome region (see Simulated sperm datasets with increased crossovers) for haplotype construction and crossover detection using reduced hetSNPs. We randomly sampled 10%, 5% and 1% of from the original list of 20K hetSNPs in the 5M regions and performed phasing and crossover calling using the downsampled lists of hetSNPs. Crossovers were called by applying *sgcocaller xo* with options "`--minDP 2 --maxDP 10 --thetaREF 0.1 --thetaALT 0.9 --cmPmb 1`", and the resulted haplotype segments were not further filtered. We observed that more crossovers were missed when the sparsity of hetSNPs increased (Fig. S11a). In addition, crossovers with shorter distances from each other were more likely to be missed. Therefore, for this simulated sperm cell dataset with the closest crossovers distances as 125k base pairs away, the number of required SNPs for a successful crossover calling result is 1,372 which corresponds to 274 SNPs per mega base region. Meiosis with more frequent and closer double crossovers would require higher density of hetSNPs.

We applied *sgcocaller autophase* on the 100 sperm cells with the reduced lists of hetSNPs for haplotype construction. *sgcocaller autophase* was run using options "`--minDP 2 --maxTotalDP 350 --chrom chr1 --maxDP 10 --minSNPdepth 5 --maxDissim 0.0099 --binSize 1000 --stepSize 800 --lookBeyondSnps 20`" for cases when using 2,727 hetSNPs and 1,372 hetSNPs. The `lookBeyondSnps` option was changed to 50 when running for

280 hetSNPs. Even when the density of hetSNPs was low, the phasing model was still able to generate the correct haplotypes (Fig.S11c).

14 Supplementary figures

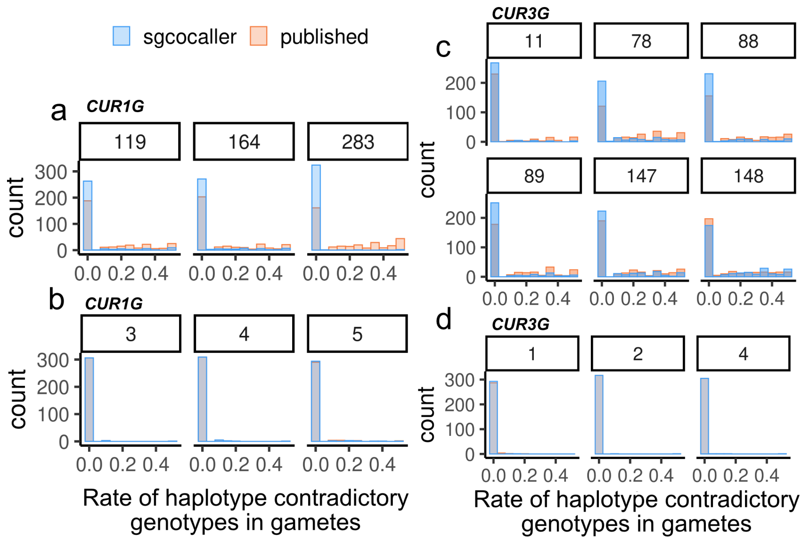

Figure S1: **SNP bins with low (and high) phasing accuracies in apricot gametes. a)** Three 100-SNP bins from CUR1G that have lower (< 0.8) phasing accuracies were identified. The rate of haplotype contradictory genotypes in SNP bins in gametes were calculate and the distribution of the fractions were plotted. **b)** Three 100-SNP bins with high phasing accuracies (>= 0.99) form CUR1G were identified and the distribution of the rate of haplotype contradictory genotypes in gametes were plotted. **c)** same as (a) but for six 100-SNP bins from CUR3G. **d)** same as (b) but for 3 bins from CUR3G.

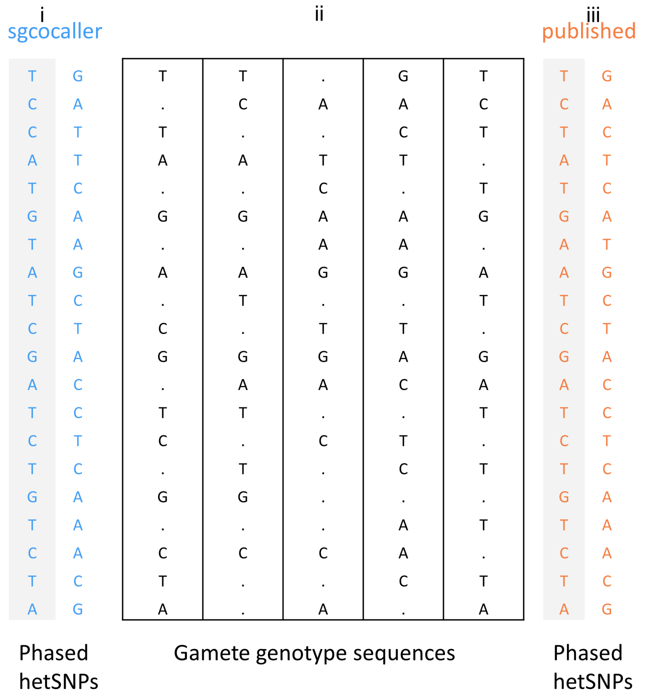

Figure S2: Representation of phased haplotypes (i and iii) and genotypes (ii) in haploid gametes

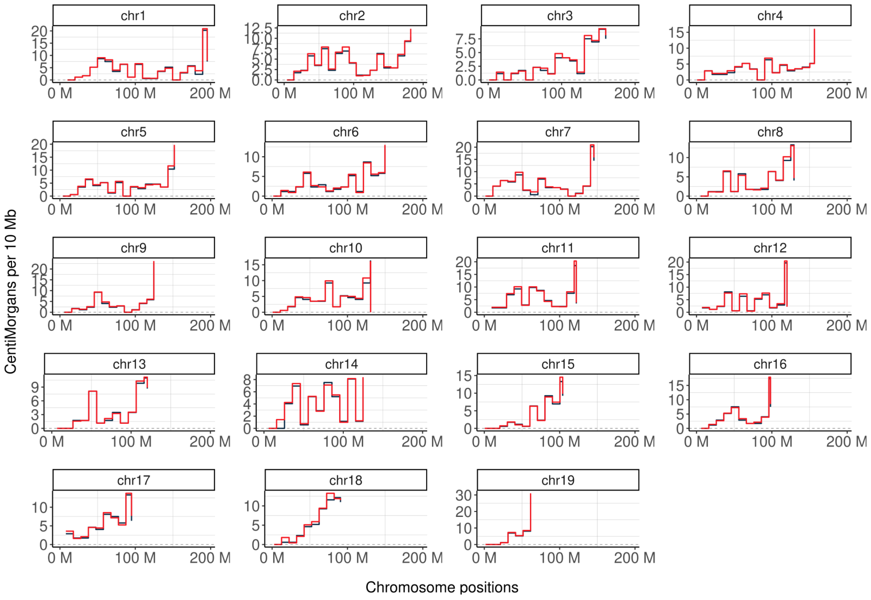

Figure S3: Genetic distances calculated by the *sgcocaller xo* and the published results from the original study in 10 Mb chromosome bins on the mouse sperm dataset for all autosomes. The same plot as in Fig5 b.

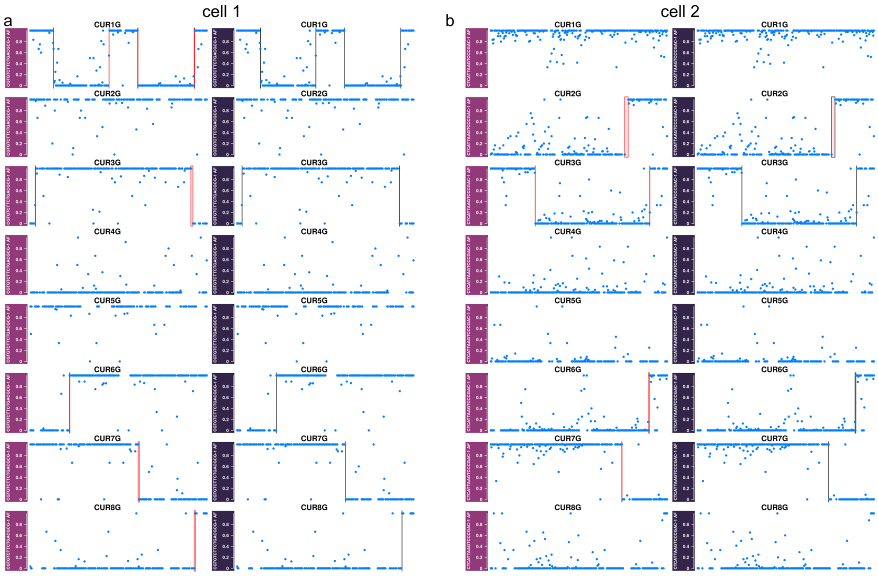

Figure S4: Crossovers called by sgcocaller-comapr workflow(left columns in a and b) and from published study (right columns in a and b) for two randomly selected apricot gametes

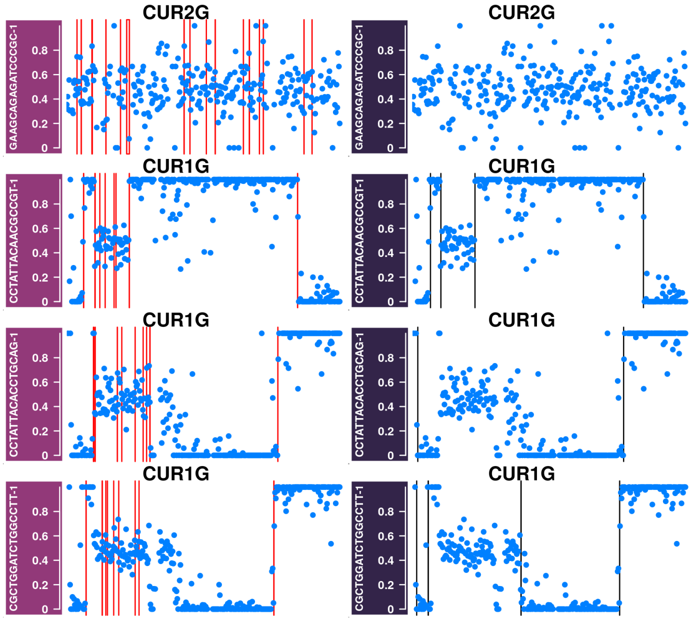

Figure S5: Examples of chromosomes with excessive crossovers called by sgco-caller xo and filtered in analysis (left column). Right column plots the crossover results released by previous study [13]

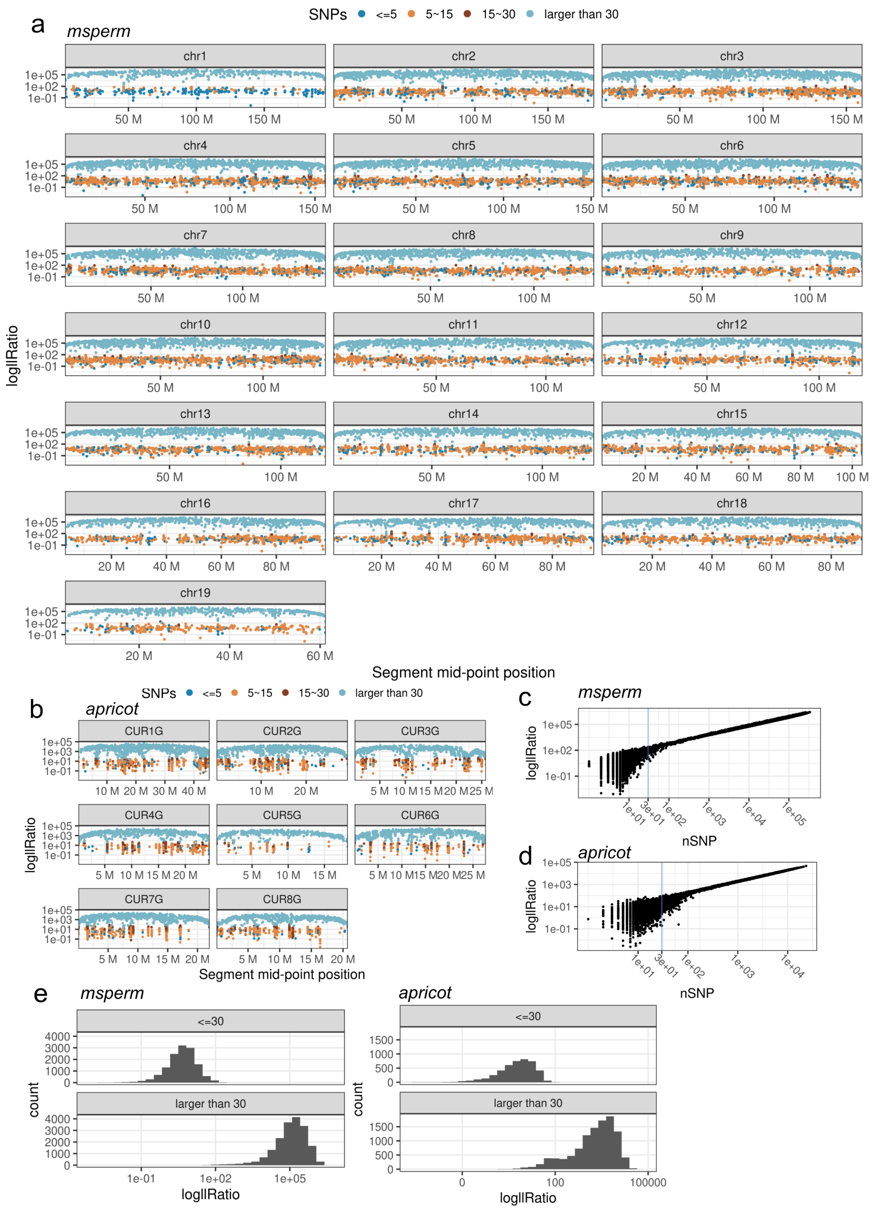

Figure S6: **False crossover filtering in different datasets a,b)** The loglIRatio of state segments and the midpoint of each segment were plotted for each chromosome in the msperm (a) and apricot dataset (b). Each point represents one segment and coloured by the number of SNPs supporting the segment.**c,d)** The number of supporting SNPs versus the loglIRatio for each state segment in the msperm (c) and the apricot dataset (d). **e)** The distribution of loglIRatio for segments in msperm and apricot dataset, grouped by whether the segments have support from more than 30 SNPs.

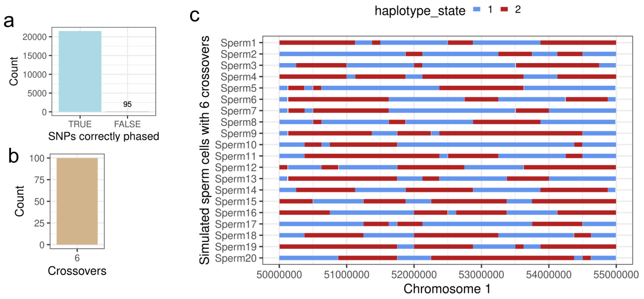

Figure S7: **Performance of sgcocaller on simulated gametes with increased crossovers a)** The number of hetSNPs that were phased correctly using the 100 simulated cells each with 6 crossovers inserted over a region of 5M base pairs. **b)** All cells were called with 6 crossovers. **c)** The haplotype segments by sgcocaller sxo for 20 representative cells were plotted and colored by the inferred haplotype states in a region of 5M base pairs (50M to 55M on chromosome 1).

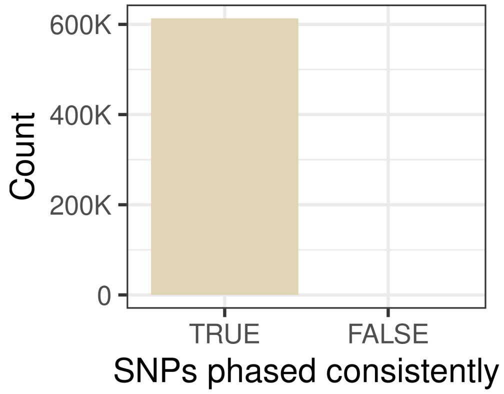

Figure S8: ***autophase* generates the same result with running phase—swphase separately.** The number of consistently phased SNPs on chromosome 3 using low coverage mouse sperm cells by *autophase* and by running *phase—swphase* separately were counted. All SNPs were phased with the same results by the two workflows.

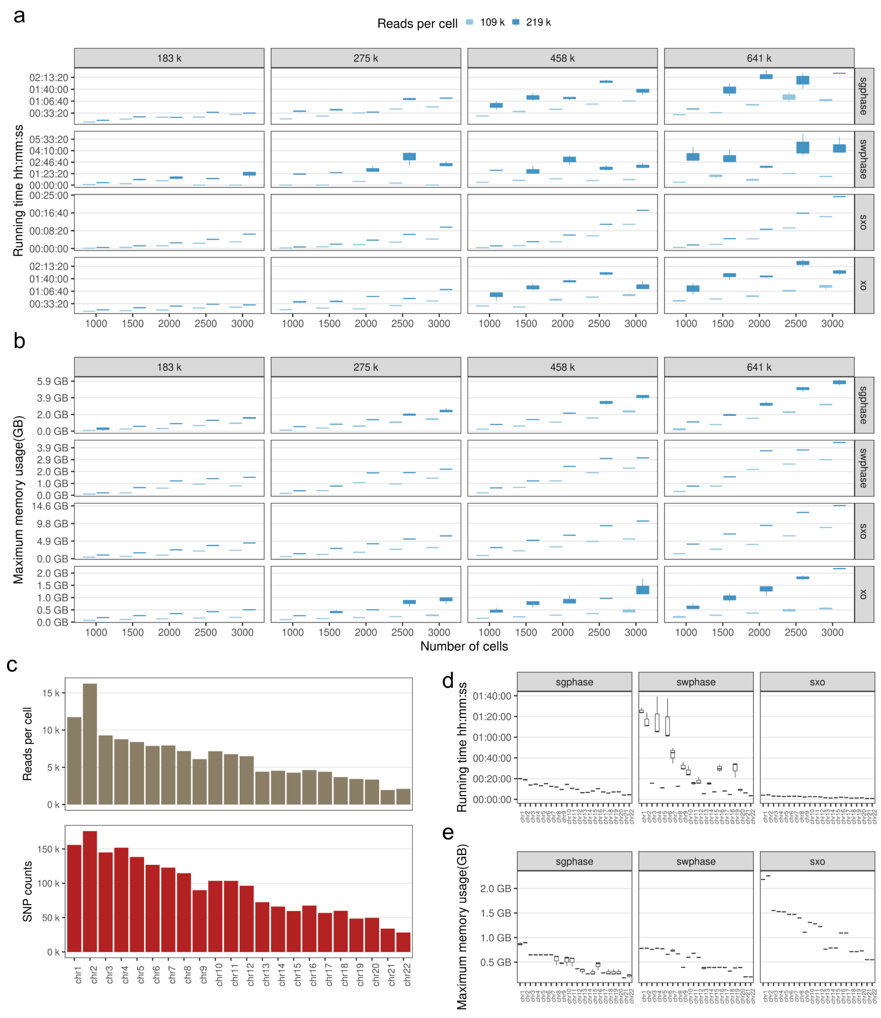

Figure S9: **Module running time and memory usage by *sgcocaller* on datasets with varying sizes a)** The running time of each module was plotted in format of hour:minute:seconds on datasets with varying numbers of SNPs (columns), numbers of cells, and mean numbers of DNA reads per cell to process. **b)** Same as a) but the maximum memory usage of each module was plotted in units of GB. **c)** The number of hetSNPs per chromosome and the mean number of reads per cell in the sperm cells from donor 1 obtained from study [9]. **d,e)** The running time of each module (d) and the maximum memory usage in units of GB (e) when processing the sperm cells from donor 1 in study [9] was plotted. Each measurement has been repeated three times by using the 'benchmark' function from `Snakemake` [14].

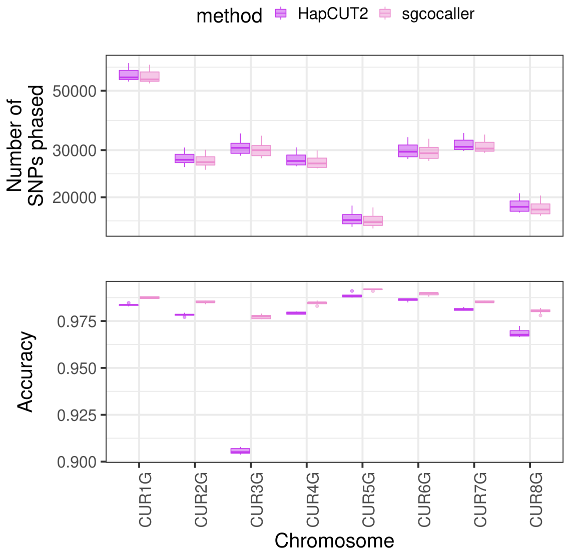

Figure S10: **Phasing performance comparison of HapCUT2 and sgcocaller** The number of phased SNPs for the apricot dataset by HapCUT2 and sgcocaller (top) and the percentage of correctly phased SNPs by HapCUT2 and sgcocaller (bottom)

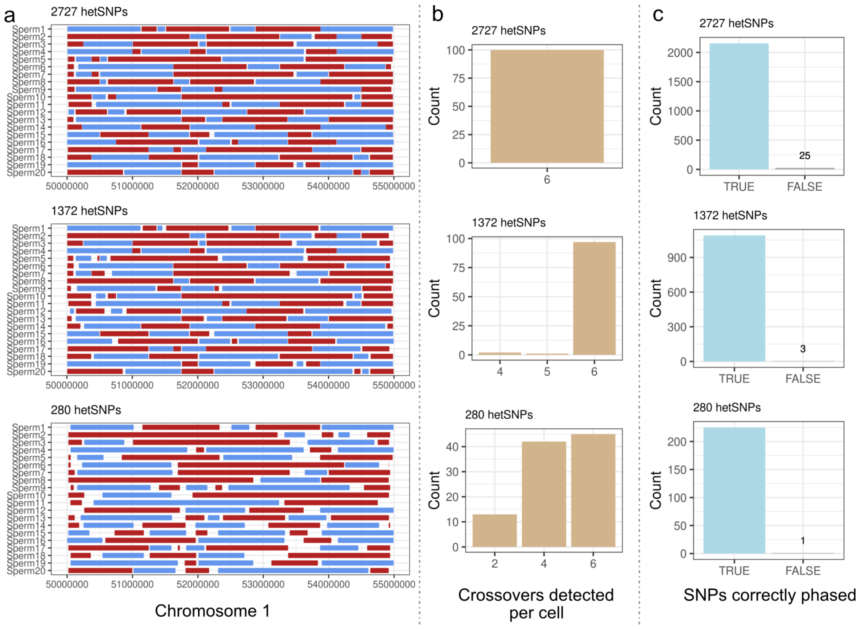

Figure S11: **The effect of low hetSNP density a)** The haplotype segments inferred by sgcocaller xo for 20 representative cells were plotted and colored by the inferred haplotype states using different numbers of hetSNPs. **b)** Number of crossovers called per cell using different number of hetSNPs. **c)** The number of (in)correctly phased SNPs using different numbers of hetSNPs.

Graph	Dataset name	Sample size	Coverage
Figure3a	apricot	367	low
Figure4	msperm	173 (after cell filtering by comapr)	high
Figure5a,b	msperm	173 (after cell filtering by comapr)	high
Figure5c,d,e	apricot	333 (the common gametes with results from three methods)	low
Figure6a	msperm_low-coverage	10 dataset repeats constructed (each with 174 or 175 cells) from 194 DNA reads downsampled mouse sperm cells	low
Figure6b	apricot	10 dataset repeats constructed (each with 330 or 331 cells) from 367 cells in total	low
Figure6c	msperm	10 dataset repeats constructed (each with 174 or 175 cells) from 194 cells in total	high
Figure6d	hsperm	11 dataset repeats constructed (each with 10 cells) from 11 human sperm cells in total	high
Figure6f,g	msperm	10 dataset repeats constructed (each with 174 or 175 cells) from 194 cells in total	high

Table S1: The dataset names and their sample sizes used in plots

References

[1] Edge, P., Bafna, V., and Bansal, V. (2017) HapCUT2: robust and accurate haplotype assembly for diverse sequencing technologies. *Genome Res.,* **27**(5), 801–812.

[2] Hinch, A. G., Zhang, G., Becker, P. W., Moralli, D., Hinch, R., Davies, B., Bowden, R., and Donnelly, P. (2019) Factors influencing meiotic recombination revealed by whole-genome sequencing of single sperm. *Science,* **363**(6433).

[3] Viterbi, A. (1967) Error bounds for convolutional codes and an asymptotically optimum decoding algorithm. *IEEE Trans. Inf. Theory,* **13**(2), 260–269.

[4] Otto, S. P. and Payseur, B. A. (2019) Crossover Interference: Shedding Light on the Evolution of Recombination. *Annual review of genetics,* **53**, 19–44.

[5] Phipson, B. and Smyth, G. K. (2010) Permutation P-values should never be zero: calculating exact P-values when permutations are randomly drawn. *Stat. Appl. Genet. Mol. Biol.,* **9**, Article39.

[6] Efron, B. and Tibshirani, R. J. (1993) An introduction to the bootstrap Chapman & Hall. *New York,* **436**.

[7] Martin, M. A. (2012) An Introduction to Bootstrap Methods with Applications to R by M.R. Chernick and R.A. LaBudde. *Aust. N. Z. J. Stat.,* **54**(2).

[8] Phipson, B. and Smyth, G. K. (2010) Permutation p-values should never be zero: calculating exact p-values when permutations are randomly drawn. *Statistical Applications in Genetics and Molecular Biology,* **9**(1), Article 39.

[9] Bell, A. D., Mello, C. J., Nemesh, J., Brumbaugh, S. A., Wysoker, A., and McCarroll, S. A. (2020) Insights into variation in meiosis from 31,228 human sperm genomes. *Nature,* **583**(7815), 259–264.

[10] Li, H., Handsaker, B., Wysoker, A., Fennell, T., Ruan, J., Homer, N., Marth, G., Abecasis, G., Durbin, R., and 1000 Genome Project Data Processing Subgroup (2009) The Sequence Alignment/Map format and SAMtools. *Bioinformatics,* **25**(16), 2078–2079.

[11] Li, H. (2018) Minimap2: pairwise alignment for nucleotide sequences. *Bioinformatics,* **34**(18), 3094–3100.

[12] Sherry, S. T., Ward, M., and Sirotkin, K. (1999) dbSNP-database for single nucleotide polymorphisms and other classes of minor genetic variation. *Genome research,* **9**(8), 677–679.

[13] Campoy, J. A., Sun, H., Goel, M., Jiao, W.-B., Folz-Donahue, K., Wang, N., Rubio, M., Liu, C., Kukat, C., Ruiz, D., Huettel, B., and Schneeberger, K. (2020) Gamete binning: chromosome-level and haplotype-resolved genome assembly enabled by high-throughput single-cell sequencing of gamete genomes. *Genome Biol.,* **21**(1), 306.

[14] Mölder, F., Jablonski, K. P., Letcher, B., Hall, M. B., Tomkins-Tinch, C. H., Sochat, V., Forster, J., Lee, S., Twardziok, S. O., Kanitz, A., Wilm, A., Holtgrewe, M., Rahmann, S., Nahnsen, S., and Köster, J. (2021) Sustainable data analysis with Snakemake. *F1000Res.,* **10**(33), 33.
